# Nanoodor Particles Deliver Drugs to Central Nervous System via Olfactory Pathway

**DOI:** 10.1002/advs.202408908

**Published:** 2025-02-25

**Authors:** Wei Zhang, Xingwang Ji, Qianyanqiu Zhao, Jinyao Qi, Wen Guo, Gaoshuo Zhang, Yujing Guan, Shenglong Li, Yuling Mao

**Affiliations:** ^1^ Department of Pharmaceutics School of Pharmacy Shenyang Pharmaceutical University 103 Wenhua Road Shenyang Liaoning 110016 P. R. China; ^2^ Second Ward of Bone and Soft Tissue Tumor Surgery Cancer Hospital of Dalian University of Technology Cancer Hospital of China Medical University Liaoning Cancer Hospital & Institute Shenyang Liaoning 110042 China; ^3^ The Liaoning Provincial Key Laboratory of Interdisciplinary Research on Gastrointestinal Tumor Combining Medicine with Engineering Shenyang Liaoning 110042 China; ^4^ Institute of Cancer Medicine Faculty of Medicine Dalian University of Technology No.2 Linggong Road, Ganjingzi District Dalian Liaoning 116024 China

**Keywords:** Agomelatine, Brain targeting, Central nervous system disorders, Nanoodor particles, Olfactory pathway

## Abstract

Central nervous system (CNS) disorders confront significant challenges in drug delivery due to the blood–brain barrier (BBB). Inspired by the rapid and precise binding of odor molecules to olfactory receptors (ORs), this research uses thiolated HPMA to construct odor nanoparticles (nanoodors) capable of delivering drugs to the CNS via the olfacto–cerebral pathway to overcome the delivery obstruction. The nanoodor core is used to encapsulate agomelatine (AGO), a CNS‐targeting antidepressant, and the encapsulation efficiency exceeded 80%. A series of thiol‐presenting nanoscale structures with different surface densities of thiol groups are constructed, and the effectiveness positively correlated with the density of thiol groups on their surface. Notably, the nanoodors enable precise brain‐targeted delivery, outperforming commercially available oral formulations in terms of drug accumulation in the brain and antidepressant effects. The study of the nanoodor transport and action mechanisms revealed that after binding to ORs, the nanoodors are rapidly delivered to the brain via the olfactory pathway. Nanoodors, the first design to deliver CNS drugs via the olfactory pathway by mimicking natural smells for the treatment of CNS disorders, are expected to achieve clinical transformation, benefiting human health.

## Graphical abstract

This study have elucidated the brain‐targeted delivery pathways and transport mechanisms of nanoodors by various technical means and found that the olfactory pathway is main one. Nanoodors can rapidly deliver drugs to the central nervous system via olfactory pathway and will hopefully solve the delivery challenges for various types of central nervous system drugs.

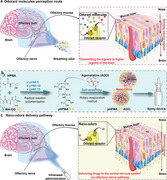

## 1 Introduction

Central nervous system (CNS) disorders affect millions of people worldwide and are therefore a global public health challenge.^[^ ^1^, ^2^ ^]^ One of the greatest obstacles in treating CNS disorders is the ineffective delivery of drugs to the brain, which mainly stems from the blood–brain barrier (BBB), a physiological barrier that protects the CNS by limiting the entry of most drugs.^[^ ^3^, ^4^, ^5^ ^]^ Although numerous technologies and drug delivery approaches have been developed to solve these problems,^[^ ^6^, ^7^, ^8^, ^9^, ^10^ ^]^ new material design perspectives are still needed to achieve the reliable and efficient delivery of drugs to the CNS and anticipated clinical impact.

Learning from nature can inspire the design of advanced materials. Our ability to rapidly sense and distinguish hundreds of odorants arises from their recognition by olfactory receptors (ORs), which subsequently get activated and transfer signals to the brain.^[^ ^11^, ^12^ ^]^ This rapid and precise process of recognition and transduction between odors and ORs inspired us to exploit this natural route to deliver drugs. ORs are G‐protein‐coupled receptors (GPCRs), which are multipass transmembrane proteins.^[^ ^13^, ^14^ ^]^ In addition to activating olfactory receptor neurons (ORNs) to generate electrical signals, they are also responsible for the transcellular transport of drugs.^[^ ^15^, ^16^ ^]^ Taking this inspiration, an odors‐like platform is on the cards to deliver drugs to the CNS by mimicking the interaction of odorant molecules with ORs. Among thousands of smells, methanethiol groups have a very low olfactory threshold and are functional groups with very high odor activity values due to the extreme sensitivity of thiol groups to ORs.^[^ ^17^, ^18^, ^19^ ^]^ We are the first to propose constructing nanoodors with surface‐presenting thiol groups to load drugs, aiming to achieve brain‐targeted drug delivery via intranasal administration.

Nose‐to‐brain delivery offers direct access to the brain bypassing the BBB, increasing brain bioavailability, and peripherally reducing unwanted side effects.^[^ ^20^ ^]^ Nanocarriers are widely used as drug delivery agents to improve drug biodistribution and targeting. Self‐assembled nanocarriers can increase drug solubility and retention time.^[^ ^21^ ^]^ When combined with nose‐to‐brain delivery, nanocarriers can be engineered for controlled release^[^ ^22^ ^]^ and effectively increase the bioavailability of CNS‐targeting drugs.^[^ ^23^, ^24^, ^25^ ^]^ HPMA is a highly biocompatible, nonimmunogenic, nontoxic, and structurally modifiable compound for specific applications.^[^ ^26^, ^27^ ^]^ Hydrophobically modified HPMA polymers spontaneously form self‐assembled nanocarriers in aqueous environments.^[^ ^28^ ^]^ Therefore, thiol groups are attached to HPMA to design a nanoodor brain‐targeted delivery material.

**Figure 1:**
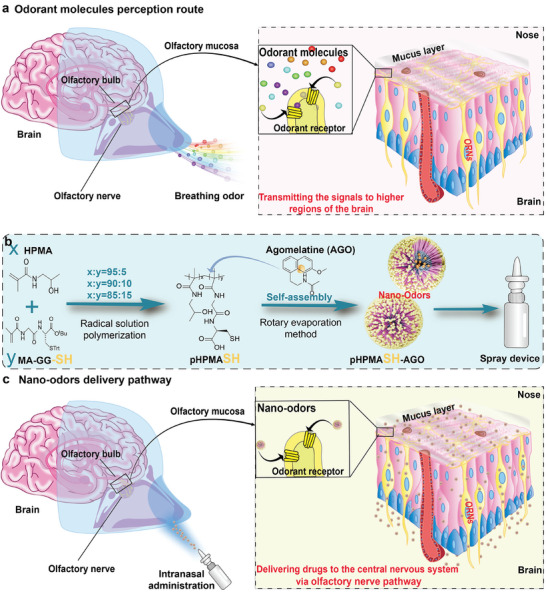
Schematic diagrams of biomimetic nanoodors design. (a) Odorant molecules perception route: after odorant molecules bind to an olfactory receptor, the interaction between the odor molecule and the olfactory neuron is converted into an electrical signal that is transmitted to the brain. (b) Synthesis process of pHPMASH‐AGO. (c) Nanoodors delivery pathway: after binding to olfactory receptors in the olfactory region, nanoodors are transmitted to the brain along the olfactory nerve pathway to deliver drugs to CNS.

Agomelatine (AGO) is an antidepressant with a low oral bioavailability due to its low solubility and significant first‐pass effect.^[^ ^29^, ^30^ ^]^ Therefore, increasing AGO accumulation in the brain is a critical clinical requirement. Herein, we constructed HPMA‐based self‐assembled nanoodor particles (nanoodors) with surface‐presenting thiol groups and used them as vehicles for AGO delivery to the brain via intranasal administration, examining the effects of the surface density of these groups to explore the key impact of smell‐mimicking behavior on the delivery process (**Figure** **1**). The study validates the theory of nano odor particles (nanoodors) as a new delivery concept through a clear explanation of the transport process and mechanism and provides a new direction for the design of brain‐targeted precision delivery carriers.

## 2 Results and Discussion

### 2.1 Preparation and Characterization of AGO‐Loaded Nanoparticles

**Figure 2:**
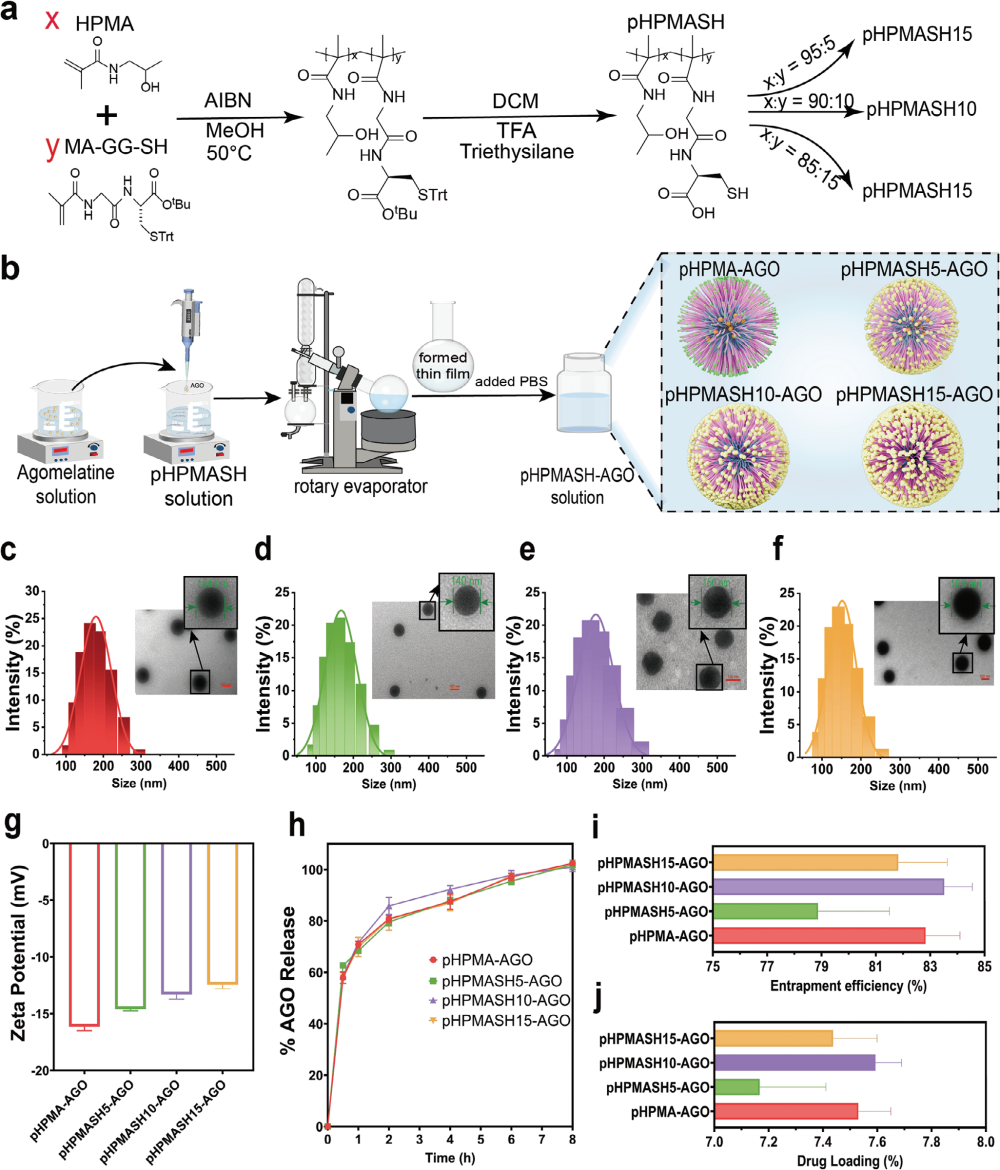
Preparation method and characterization of self‐assembled nanoparticles. (a) Shorthand for HPMA modified with different proportions of thiol groups. (b) Schematic diagram of the preparation method. Particle sizes and TEM images of (c) pHPMA‐AGO, (d) pHPMASH5‐AGO, (e) pHPMASH10‐AGO, and (f) pHPMASH15‐AGO. (g) Zeta potential of different nanoparticles. (h) In vitro release profiles of different nanoparticles.(i) Entrapment efficiency of AGO in different nanoparticles. (j) Drug loading of AGO in different nanoparticles.

Self‐assembled nanoparticles with thiol group–modified surfaces were prepared by the free‐radical polymerization of HPMA and thiolated *N*‐methacryloyl‐glycylglycine (MA‐GG‐SH) (**Figures** **2**a and S1–S3, and Table S1, Supporting Information). The resulting polymer (pHPMASH) was self‐assembled into nanoparticles using rotary evaporation, and the lipophilic nanoparticle core was used to encapsulate AGO (Figure 2b). Dynamic light scattering analysis indicated that the diameter of the AGO‐loaded nanoparticles (pHPMASH‐AGO) minimally increased with the increasing surface density of thiol groups but remained below 200 nm (Figure 2c–f). Transmission electron microscopy (TEM) imaging (Figure 2c–f) revealed that these nanoparticles were spherical or near‐spherical, had a uniform size distribution, and did not exhibit aggregation. Particles of such size favor rapid drug transit through the mucosal layer to achieve high delivery efficiencies. MA‐GG‐SH was incorporated at three different feed ratios (5%, 10%, and 15%) to afford pHPMASHx‐AGO (x = feed ratio of MA‐GG‐SH in mol%) nanoparticles with varying negative charge densities (Figure 2g).

The incorporation of thiol groups did not adversely affect drug release, which indicated the successful preparation of self‐assembled nanoparticles capable of enhancing the therapeutic efficacy of AGO (Figure 2h). The drug encapsulation efficiency (EE) and loading (DL) were quantified using a standard curve constructed using the absorbance of AGO at 303 nm (Figure S4, Supporting Information). The EEs of the pHPMA‐AGO, pHPMASH5‐AGO, pHPMASH10‐AGO, and pHPMASH15‐AGO nanoparticles were 82.8 ± 1.26%, 78.9 ± 2.93%, 83.5 ± 1.13%, 81.8 ± 1.93%, respectively, and the corresponding DLs were 7.53 ± 0.12%, 7.17 ± 0.27%, 7.59 ± 0.10%, 7.44 ± 0.18%, respectively (Figure 2i,j, Table S2).

**Figure 3:**
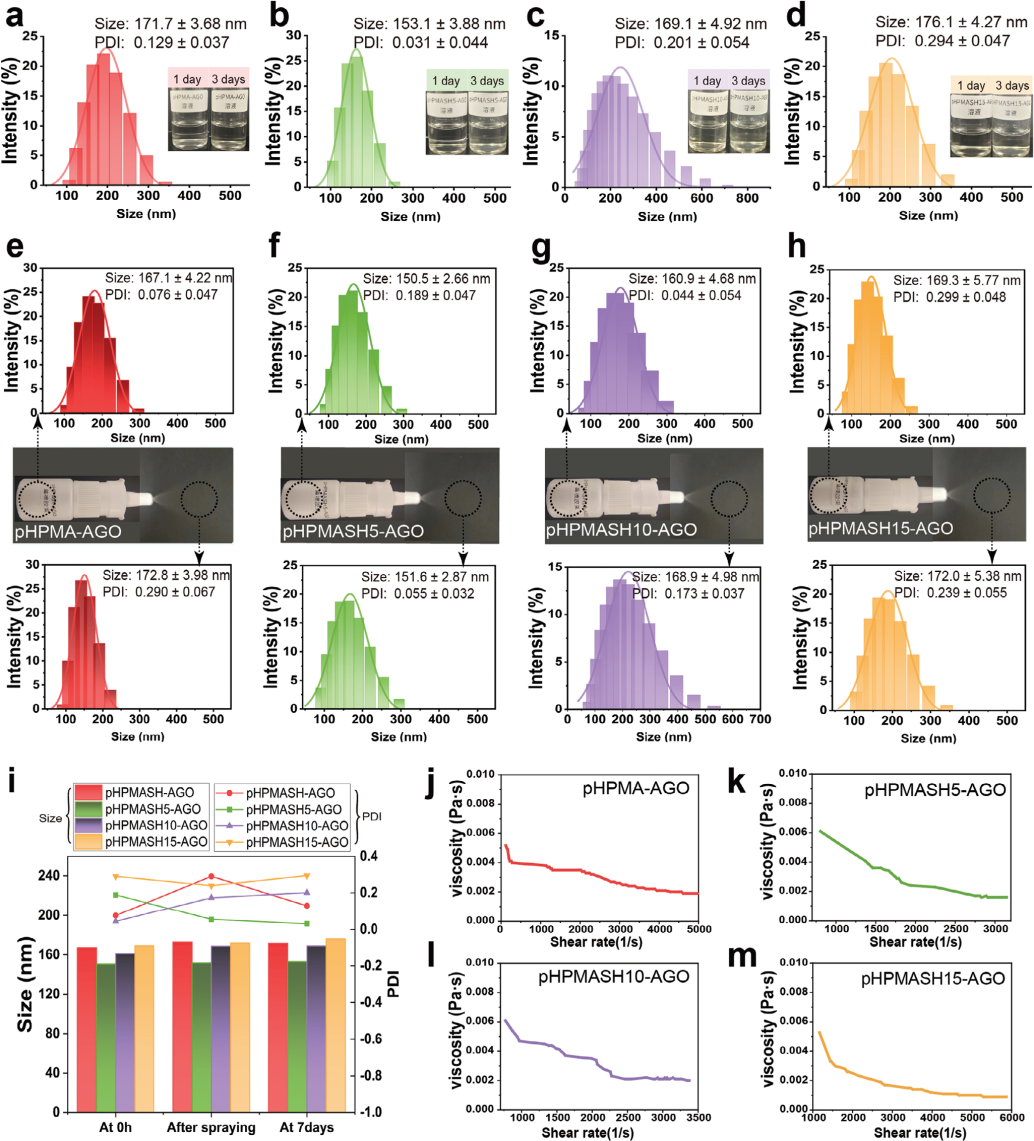
Properties and spray properties of self‐assembled nanoparticles. Particle sizes and the images of (a) self‐assembled nanoparticles, (b) pHPMASH5‐AGO, (c) pHPMASH10‐AGO, and (d) pHPMASH15‐AGO after 3 days. Particle sizes of (e) pHPMA‐AGO, (f) pHPMASH5‐AGO, (g) pHPMASH10‐AGO, and (h) pHPMASH15‐AGO after spraying and at the completion of preparation. (i) Particle size and PDI changes of self‐assembled nanoparticles after spraying, after being left for 3 days, and at the completion of preparation. (j) Rheological property of pHPMA‐AGO, (k) pHPMASH5‐AGO, (l) pHPMASH10‐AGO, and (m) pHPMASH15‐AGO.

The sustained release of AGO from the self‐assembled nanoparticles under physiological conditions highlighted the possibility of a prolonged therapeutic efficacy (Figure 2h). Subsequently, we evaluated the stability of the self‐assembled nanoparticles and their suitability for nasal spray formulations, revealing that three‐day storage at 4 °C did not cause significant color or appearance changes (**Figure** **3**a–d) and minimally affected the particle size and polydispersity index (PDI). These results suggested that the nanoparticles were stable under refrigeration and minimally affected the particle size and polydispersity index (PDI). These results suggested that the nanoparticles were stable under refrigeration. The AGO‐loaded nanoparticles were also tested in a nasal spray device that produced a uniform mist upon spraying. Spraying had a negligible effect on particle size, and the nanoparticles remained well‐dispersed (Figure 3e–i). This behavior indicated that the shear forces generated by the delivery device had a negligible impact on the nanoparticle structure, confirming the stability of the formulation under the employed conditions. The viscosity of the prepared solution exhibited a minimal dependence on the shear rate (Figure 3j–m).

### 2.2 Evaluation of Olfactory Mucosa Crossing Ability

**Figure 4:**
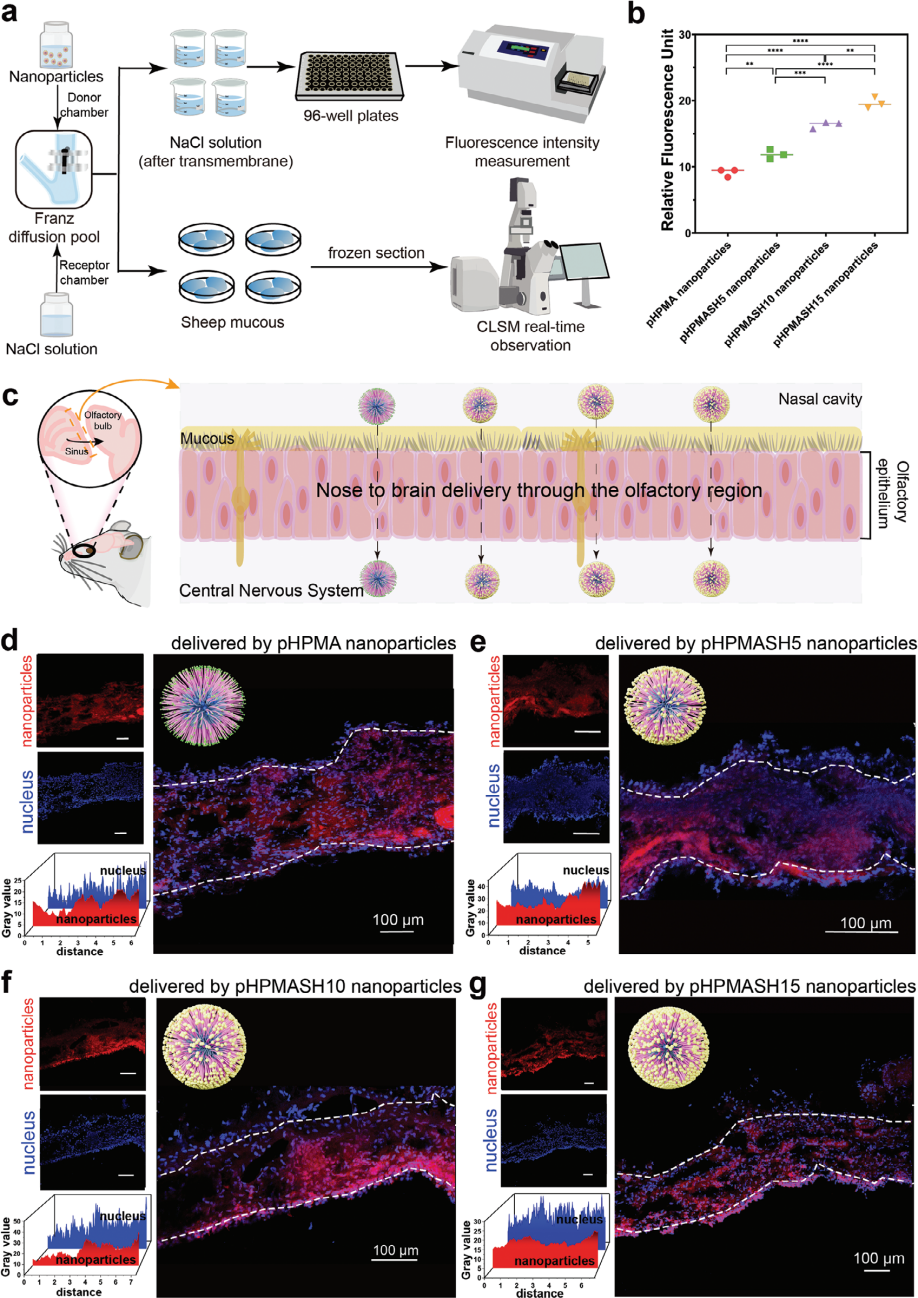
Studies of transmembrane conditions. (a) Schematic diagram of experimental design. (b) Fluorescence intensity of the solution in the lower layer of the diffusion cell after transmembrane crossing. (c) Schematic diagram of mucosa. Fluorescence pictures of mucous after transmembrane crossing under the confocal microscope of (d) pHPMA nanoparticles, (e) pHPMASH5 nanoparticles, (f) pHPMASH10 nanoparticles, and (g) pHPMASH15 nanoparticles. Results are presented as mean ± SD (*n* = 3). **P < 0.01, ***P < 0.001, ****P < 0.0001. One‐way ANOVA with a Tukey post‐hoc analysis was used for comparison among multiple groups.

To explore the transmembrane capabilities of different nanoparticles, we studied their ability to penetrate the nasal mucosa (**Figure** **4**a). The nucleus of olfactory epithelial cells was stained with DAPI, which exhibits blue fluorescence. Cyanine7 amine (Cy7), a dye known for its near‐infrared fluorescence emission and represented in red in our images, was used to visualize drug penetration. The fluorescence intensity in the administration reservoir was normalized prior to testing. After the completion of the permeation test, we measured the fluorescence intensity of the receiving fluid. For all nanoparticle types, the fluorescence intensity in the receptor pool exceeded that in the Cy7 solution and was highest for the pHPMASH15 nanoparticles (Figure 4b). This result demonstrated that our nanoparticles facilitated the transport of poorly soluble drugs across the olfactory epithelium (OE) and indicated that the surface density of thiol groups was positively correlated with the permeation degree.

Figure 4c presents a schematic of the olfactory mucosa. The combined fluorescence from DAPI and Cy7 highlighted the significant penetration of the nanoparticles through the OE, as evidenced by the intense red and blue signals observed in the olfactory regions (Figure 4d–g). The red fluorescence of Cy7 was detected not only on the surface but also within the inner regions of the OE, illustrating the effectiveness of nanoodor transport across the olfactory epithelium (Figure 4d–g). These results provide a basis for future studies.

### 2.3 Nanoodor Accumulation in the Brain Examined by Optical Imaging

**Figure 5:**
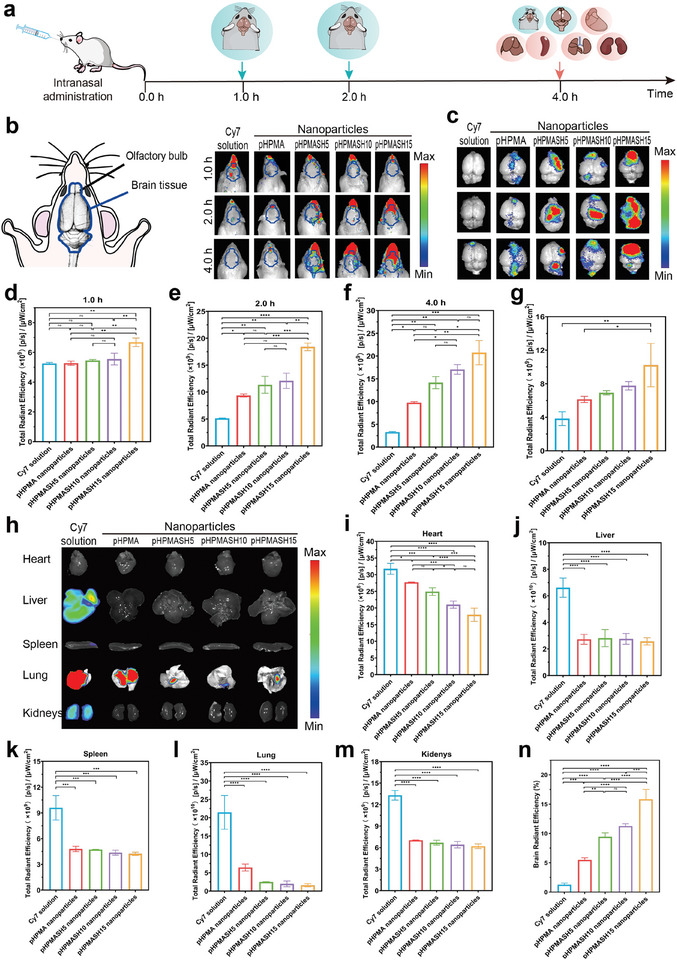
In vivo and ex vivo studies of biodistribution. (a) Experimental design for administration. (b) Location of brain tissue in the rat and real‐time fluorescence imaging of rats after intranasal administration of Cy7‐loaded nanoparticles at 1 h, 2 h, 4 h post after intranasal administration. (c) Ex vivo imaging of the brain tissues after intranasal administration of Cy7‐loaded nanoparticles. *n* = 3 per group. Corresponding fluorescence analysis of rats at (d) 1 h, (e) 2 h, and (f) 4 h post after intranasal administration of Cy7‐loaded nanoparticles. (g) Corresponding fluorescence analysis of the brain tissues after intranasal administration of Cy7‐loaded nanoparticles.(h) Ex vivo imaging of the main tissues after intranasal administration of Cy7‐loaded nanoparticles. *n* = 3 per group. Corresponding fluorescence analysis of (i) heart, (j) liver, (k) spleen, (l) lungs, (m) kidneys after intranasal administration of Cy7‐loaded nanoparticles. (n) Brain tissue fluorescence intensity as a percentage of the fluorescence intensity sum of the six major tissues by combining brain tissue fluorescence intensity. Results are presented as mean ± SD. *P < 0.05, **P < 0.01, ***P < 0.001, ****P < 0.0001 (ns: no significance). One‐way ANOVA with a Tukey post‐hoc analysis was used for comparison among multiple groups.

**Figure** **5**a shows the results of real‐time fluorescence imaging and distribution of nanoparticles in the rat brain after intranasal administration. Considering the potential of thiol modification to enhance the brain‐targeting capabilities of the pHPMA nanoparticles, we investigated the accumulation of the pHPMASH nanoparticles in the brain in vivo and in vitro. The nanoparticles were loaded with Cy7, a poorly soluble near‐infrared fluorescent dye that facilitates imaging. A nasally administered Cy7 solution served as a control. In vivo imaging was performed at 1.0, 2.0, and 4.0 h postadministration using an in vivo imaging system (Figure 5b).

Ex vivo imaging was performed to identify brain regions (Figure 5c). The fluorescence intensities of nanoparticle groups exceeded that of the control group at 1 h post administration. However, the advantage of the thiol groups was not fully evident at this point (Figure 5d). The fluorescence intensity of the control group peaked at 1.0 h, whereas that of the nanoparticle groups continued to increase beyond this point. This behavior suggested that the formation of self‐assembled nanoparticles significantly enhanced drug accumulation and retention in the brain. At 2.0 h, the fluorescence intensity in the nanoparticle groups markedly exceeded that in the control group. The fluorescence intensity increased with the increasing thiol group density. Specifically, the fluorescence intensity of the pHPMASH15 nanoparticles was nearly four times that of the Cy7 solution and almost twice that of the unmodified pHPMA nanoparticles (Figure 5e) and remained at this level at 4 h (Figure 5f). The in vitro fluorescence intensities of pHPMASH15, pHPMASH10, pHPMASH5, and pHPMA nanoparticles were 2.66, 2.02, 1.80, and 1.60 times that of the free Cy7, respectively. In vitro brain analysis confirmed that fluorescence intensity (and hence, the degree of accumulation in the brain) increased upon the introduction of thiol groups and was positively correlated with their surface density (Figure 5g). These results suggested that (i) the encapsulation in the nanoodors significantly enhanced drug accumulation and retention in the brain and (ii) the incorporation of thiol groups enhanced the nanoodor ability to transport drugs across the OE to the brain.

### 2.4 Ability of Intranasally Administered Nanoodors to Reduce Systemic Tissue Distribution

The distribution of the self‐assembled nanoparticles in different tissues was evaluated after intranasal administration. At 4 h post administration, the tissues were collected and subjected to fluorescence analysis (Figure 5h). Markedly weak signals were observed in the heart, liver, lung, spleen, and kidneys in nanoparticle groups, whereas significantly strong signals were observed for the Cy7 solution administered via the same route (Figure 5i–m). The fluorescence intensity in tissues other than the brain was inversely proportional to the surface density of the thiol groups. The reduced distribution in other tissues not only indicated that the nanoodors could minimize systemic side effects but also indirectly suggested increased accumulation in the brain.

Subsequently, we quantified the proportion of fluorescence intensity in the brain tissue relative to the total tissue fluorescence (Figure 5n), showing that for the Cy7 solution, this proportion was remarkably lower than those in the nanoparticle groups. The brain fluorescence intensity ratios in the pHPMASH15, pHPMASH10, and pHPMASH5 groups were approximately nearly three‐, two‐, and nearly two‐fold higher, respectively, than that observed for the pHPMA nanoparticles. These results not only indicated that the self‐assembled nanoparticles increased the time of drug retention in the brain but also confirmed that with the increasing thiol group density, the brain‐targeting capabilities of the nanoodors were enhanced and accumulation in other body parts was reduced.

### 2.5 In Vivo Safety Evaluation

The safety of the formulation was assessed in vivo by a comparative study involving different control groups and histological examinations. Nasal mucosa treated with isopropanol and saline served as positive and negative controls, respectively. Tissue sections were stained with hematoxylin and eosin and examined under a light microscope. After intranasal administration, the nasal mucosa in the saline and formulation groups showed well‐preserved epithelial structures with no detectable signs of damage, inflammatory cell infiltration, hemorrhage, necrosis, or edema (Figure S5a,c–f, Supporting Information). The isopropanol group exhibited disrupted epithelial integrity and inflammatory cell infiltration, which was indicated by the blue arrow (Figure S5b, Supporting Information). Thus, the nanoodors were concluded to be safe for intranasal administration, representing a viable option for applications that can direct nasal delivery without compromising mucosal integrity.

### 2.6 Effect of Nanoodors on Amount of AGO in Brain Revealed by Pharmacokinetic Analysis

**Figure 6:**
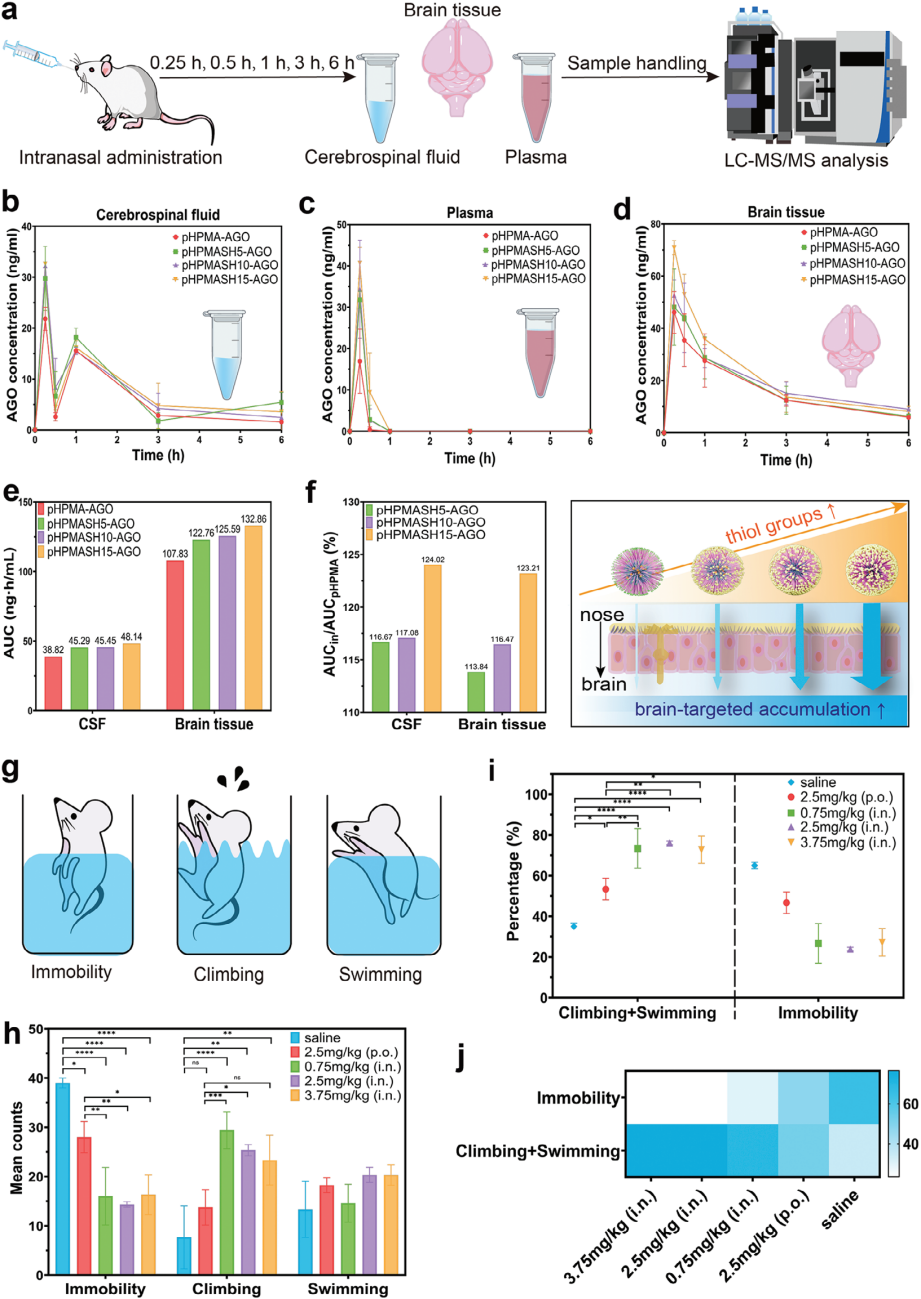
Pharmacokinetic studies and pharmacodynamics in rats after intranasal administration. (a) Schematic diagram of the pharmacokinetic studies. (b) Cerebrospinal fluid, (c) plasma, and (d) whole brain tissue concentrations of AGO in rats after intranasal administration of different formulations of self‐assembled nanoparticles at doses equivalent to AGO at 0.25 h, 0.5 h, 1 h, 3 h, and 6 h. e) The area under the curve (AUC) of pHPMA‐AGO, pHPMASH5‐AGO, pHPMASH10‐AGO, and pHPMASH15‐AGO groups. (f) AUC_in_/AUC_pHPMA_ of pHPMASH5‐AGO, pHPMASH10‐AGO, and pHPMASH15‐AGO groups. Schematic diagram: the higher the density of thiol groups on the surface of nanoodors, the higher the accumulation of brain targeting. (g) Schematic diagram of the rat immobile, swimming, and climbing. (h) Mean counts of immobility, climbing, and swimming. (i) Immobility as a percentage of total time in each group, and adding swimming and climbing time to the mix. (j) The analysis of behaviors during the modified MFST. Results are presented as mean ± SD (*n* ≥ 3). *P < 0.05, **P < 0.01, ***P < 0.001, ****P < 0.0001 (ns: no significance). One‐way ANOVA with a Tukey post‐hoc analysis was used for comparison among multiple groups.

Using LC‐MS/MS, we analyzed the concentrations of AGO in the brain tissue, CSF, and plasma collected 0.25, 0.5, 1, 3, and 6 h after intranasal administration (**Figure** **6**a). Similar characteristic peaks of AGO were observed in the whole brain tissue, cerebrospinal fluid (CSF), and plasma, demonstrating that AGO was efficiently detected in these samples (Figure S6, Supporting Information). The areas under the concentration–time curve (AUC) was shown in Figure 6b–d. Pharmacokinetic parameter calculations revealed that peak concentrations in the CSF, brain tissue, and plasma were reached at 15 min post administration. The surface density of thiol groups significantly influenced pharmacokinetic results and was positively correlated to peak concentrations in all samples (Figure S7, Supporting Information). The same trend was observed for AUC (Figure 6e). The AUC_0‐∞_ values of the pHPMASH5‐AGO, pHPMASH10‐AGO, and pHPMASH15‐AGO formulations were 116.67%, 117.08%, and 124.02% that of the pHPMA‐AGO control, respectively (Figure 6f), indicating enhanced drug delivery and retention. A similar trend was observed for the brain tissue. The surface density of thiol groups was positively correlated with the accumulation of AGO in the brain.

Additionally, we assessed the pharmacokinetic profile of orally (p.o.) administered AGO at various time points (Figure S8, Supporting Information) and compared it with that of intranasally (i.n.) administered AGO. Despite the oral dose exceeding the intranasal dose 3.3‐fold, the AUC_0‐∞_ and C_max_ values of the pHPMASH5‐AGO (i.n.), pHPMASH10‐AGO (i.n.), and pHPMASH15‐AGO (i.n.) groups significantly exceeded those observed for orally administered AGO, highlighting the enhanced bioavailability and brain targeting ability of the intranasal formulations (Table S3, Supporting Information).

### 2.7 Effects of Nanoodors on Depressive Behavior Revealed by Pharmacodynamic Analysis

Based on the above results, we selected the pHPMASH5 group, which demonstrated the highest brain accumulation, for pharmacodynamic analysis. The pharmacodynamics results were tested by a modified forced swimming test (MFST), scoring is based on two types of active behaviors, swimming and climbing, and the use of a time sampling technique, where the predominant behavior is measured in 5‐s intervals. Immobility was also a primary scoring criterion (Figure 6g). Treated animals exhibited a significantly lower immobility time than untreated ones (Figure 6h). Compared with the oral administration of AGO, intranasal delivery significantly reduced the prevalence of immobility behavior despite the intranasally administered dose being one‐third of the orally administered dose, which demonstrated the high therapeutic efficacy of the latter. The reduction in immobility time confirmed that encapsulated AGO effectively reached the site of action and exhibited antidepressant activity.

Medium and high doses did not significantly increase swimming or climbing times compared with low doses, and the immobility time at the medium dose was minimally lower than that at the low dose (Figure 6i,j). This finding was attributed to the ability of excessive AGO intake to cause CNS depression and reduce excitability. Thus, we concluded that the nanoodors substantially increased the brain‐targeted accumulation of AGO and that intranasal administration reduced the required dosage and, hence, systemic side effects.

### 2.8 Mechanism of Drug Transport to Brain by Nanoodors Across Olfactory Mucosa

**Figure 7:**
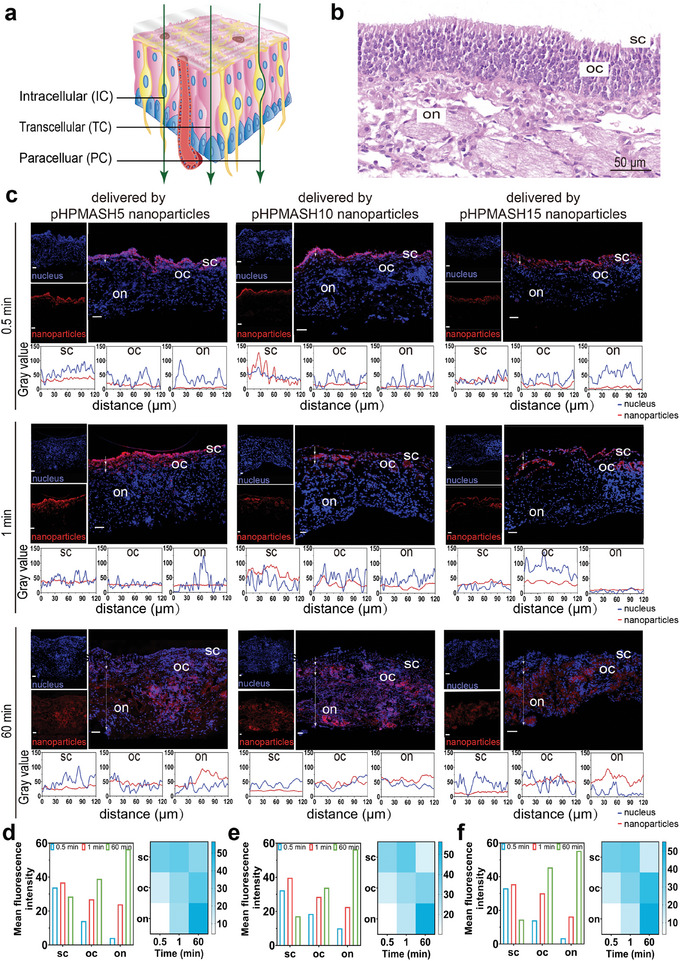
Mechanistic studies of the entry of nanoodors into the olfactory brain through the olfactory mucosal epithelial route. (a) Schematic diagram of rat olfactory pathway. (b) Epithelial structure of olfactory region. (c) Conditions of nanoodors and semiquantitative analysis in sc, oc, and on regions at 0.5 min, 1 min, and 60 min across the olfactory mucosal epithelium (scale bar: 100 µm). (d) Fluorescence intensity per unit area of drugs which delivered by pHPMASH5 nanoparticles, (e) pHPMASH10 nanoparticles, (f) pHPMASH15 nanoparticles. (on, olfactory nerve; sc, sustentacular cells; oc, nucleus of olfactory cells).

Two mechanisms have been identified for nanoodor entry into the olfactory region, namely, the olfactory mucosal epithelial route and olfactory nerve route.^[^ ^16^, ^31^, ^32^ ^]^ Olfactory filaments penetrate the nasal mucosa in the upper part of the nose and enable the intracellular transport of substances through the nerve axon (intracellularly). In addition, transport across the mucosa can occur between cells (paracellularly) or through cells (transcellularly) (**Figure** **7**a).

We observed the epithelial structure of the olfactory region (Figure 7b) and revealed a clear trend in the distribution of the drug within the mucosa at 0.5, 1, and 60 min postadministration using confocal microscopy, showing that the nanoodors progressively diffused toward the brain over time (Figure 7c). Moreover, we measured the fluorescence intensity per unit area at different time points in each group and found that this intensity in the sustentacular cells (SC), nucleus of olfactory nerve cells (OC), and olfactory nerve (ON) sites increased from 0.5 to 1 min. At 1 h, we observed a decrease in fluorescence intensity at the SC site, accompanied by an accumulation at both the OC and ON sites (Figure 7d–f). This trend, which was observed for the three groups, indicated the gradual diffusion of the nanoodors from the nose to the brain tissue and confirmed that the transmembrane process occurred via the olfactory mucosal epithelial route.

Previous studies have reported that drug delivery by the intraneuronal route is relatively slower than delivery through olfactory epithelial cells^[^ ^33^ ^]^ Therefore, the notable diffusion difference observed between 0.5 and 1 min was probably due to the olfactory mucosal epithelial route, with a possible cumulative contribution from the olfactory nerve route at the 1 h mark. These figures reveal the transmembrane process of the nanoodors.

### 2.9 Mechanism of Drug Delivery to Brain by Nanoodors Through Olfactory Nerve Route

In the three nanoodor groups, the drug‐loaded nanoparticles entered the brain through the olfactory region. Based on the above results, the pHPMASH15 nanoparticles were selected for further study.

To verify that nanoodors can bind to OR, we used a OR2C1 antibody to label OR2C1. Yellow fluorescent markers were used for the OR2C1, green fluorescent markers were used for nanoodors, and blue fluorescent markers were used for nucleus. The results showed that nanoodors were colocalized with OR2C1 (Figure S9, Supporting Information), indicating that nanoodors could be fully combined with OR.

**Figure 8:**
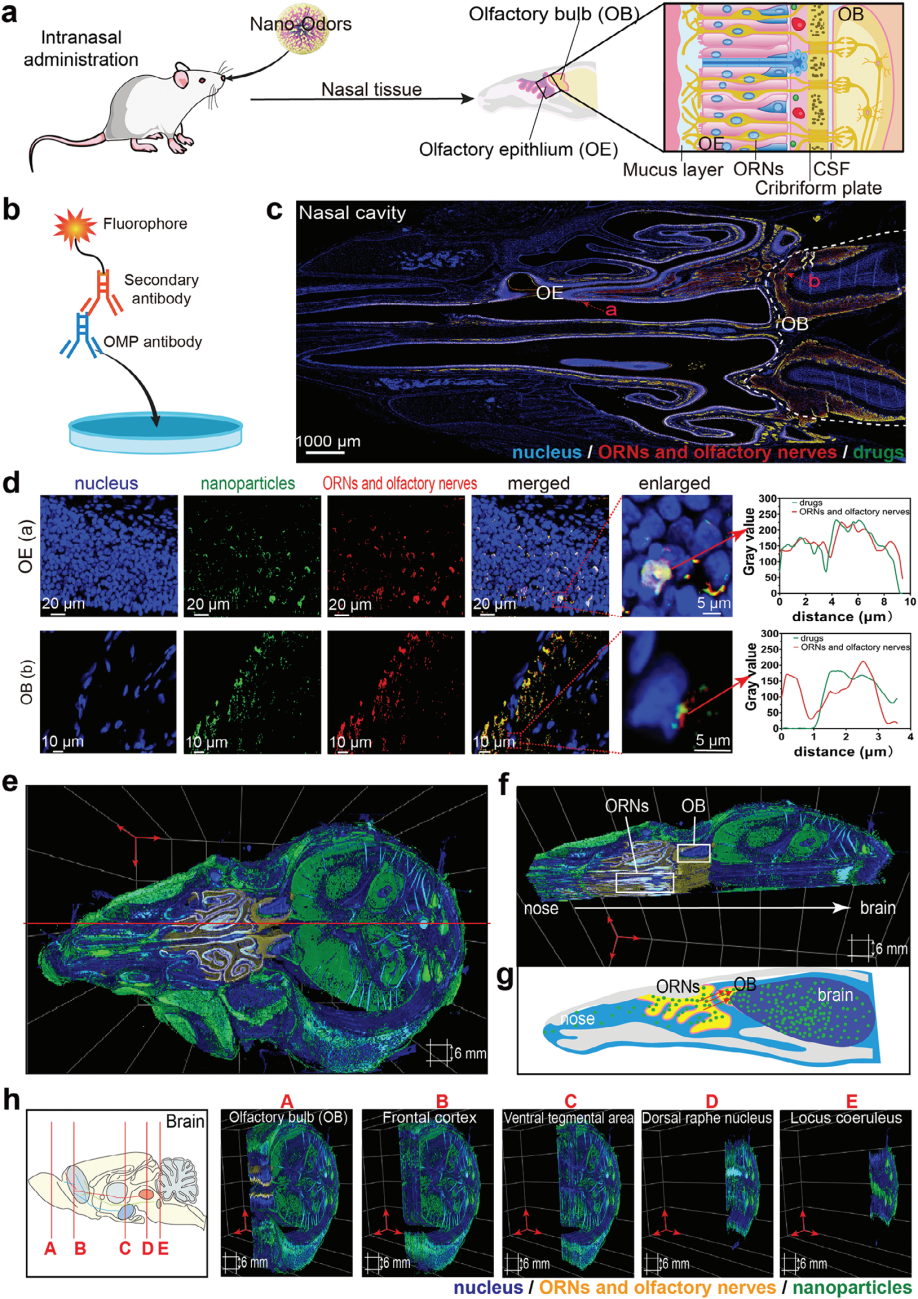
Mechanistic studies of the entry of nanoodors into the olfactory brain through the olfactory nerve route. (a) Structure diagram of the nasal region in rats. (b) Schematic diagram of OMP detection principle, fluorophore was used to label the secondary antibody. (c) 60 min after intranasal administration, an immunofluorescence dual staining image showing nanoodors (green) and the olfactory neuron marker OMP (red). (d) In the olfactory epithelium (OE) and olfactory bulb (OB) regions, the colocalization of drugs represented by Cy2 (green) and olfactory neurons labeled by ORNs and olfactory nerves (red) was determined by confocal microscopy, DAPI (blue). And colocalization magnification of OE regions and OB regions and semiquantitative results. (e) 3D structure of nose and brain tissue in rats. (f) Immunofluorescence double staining image of a drug represented by Cy2 (green) and the olfactory neuron marker OMP (yellow). The tissues were sectioned along the red line of Figure 8e. (g) Schematic diagram of drug delivery to the brain via the olfactory nerve pathway. (h) Distribution of drugs in the brain. Segmentation at five sites: (A) olfactory bulb, (B) frontal cortex, (C) ventral tegmental area, (D) dorsal raphe nucleus, and (E) locus coeruleus.

To further validate transport via the olfactory nerve route after administration in the olfactory region, we used antibodies against the olfactory neuron marker OMP. Olfactory nerves originating from ORNs in the nasal mucosal cluster pass through the cribriform plate to reach the olfactory bulb (OB) (**Figure** **8**a). Red fluorescent markers were used for the ORNs and olfactory nerves, green fluorescent markers were used for nanoodors, and blue fluorescent markers were used for nucleus (Figure 8b). Significant colocalization in both OE and OB was observed (Figure 8c). The results revealed the colocalization of Cy7 with OMP‐labeled olfactory neurons in the nasal mucosal olfactory region, and Cy7 was also detected in the olfactory nerves connecting the mucosa to the OB (Figure 8d), which confirmed nanoodor delivery through the olfactory nerve route.

In the nasal epithelium, approximately five million olfactory neurons each possess one of approximately 900 different ORs, all of which are GPCRs.^[^ ^34^ ^]^ Among the membrane proteins, GPCRs are crucial for signal transduction and material transport, thus representing important pharmaceutical targets, with their overexpression in various cell types enhancing nanoparticle delivery through ligand–receptor interactions.^[^ ^35^, ^36^, ^37^ ^]^ Herein, the nanoodors entered the OB through the olfactory region when administered intranasally. The thiol groups significantly enhanced the binding capacity of the carrier to the GPCRs on the cilia of ORNs, thereby increasing the amount of drug delivered to the OB. Furthermore, the drug could access the OB via both the olfactory mucosal epithelial route and the olfactory nerve route. In the olfactory nerve pathway, the drug was absorbed by ORNs and transmitted along the axons into the brain. In this process, GPCRs played a proactive role and facilitated the receptor‐dependent endocytosis of nanoparticles, significantly enhancing the efficiency of the nanoparticle delivery system.

### 2.10 3D Structure Visualizing Nanoodor Delivery to Brain Through Olfactory Nerve Route

Nanoodor delivery through the olfactory nerve route provides further insights into drug delivery from the nose to the brain. Figure 8e presented the 3D structure of the nasal and brain tissues in rats. Cy5 was used as a secondary antibody to label ORNs and the olfactory nerve with yellow fluorescence, whereas green fluorescence indicated the presence of a poorly soluble drug. The tissues were sectioned along the red line in Figure 8e, and distinct green fluorescence was observed in the ORNs, olfactory nerves, and olfactory region (Figure 8f). This finding confirmed that the nanoodors were delivered through the olfactory nerve route, entered the olfactory nerve cells and were axonally transported to the OB (Figure 8g).

Additionally, the brain was sectioned along the red line to obtain five sections showing dual fluorescence staining: The OB (A), frontal cortex (B), ventral tegmental area (C), dorsal raphe nucleus (D), and locus coeruleus (E). Prominent Cy2 (green) fluorescence was observed in all five sections (Figures 8h and S10, Supporting Information).^[^ ^30^ ^]^ Collectively, these results strongly indicated the capability of the nanoodors to deliver poorly soluble drugs to the brain via the olfactory nerve route. Additionally, AGO is an agonist of melatonin MT_1_ and MT_2_ receptors, which are primarily located in the pineal gland. Additionally, AGO acts as an antagonist of 5‐HT_2C_ receptors, with binding sites mainly concentrated in the amygdala, hippocampus, and prefrontal cortex of the brain.^[^ ^30^, ^38^ ^]^ In these five sections, a substantial presence of green fluorescence was observed in regions such as the prefrontal cortex, which indicated that the nanoodors could effectively deliver AGO to its target sites. This finding corroborated the reliability of the pharmacological and pharmacokinetic analysis results obtained previously.

## 3 Conclusion

Inspired by the odorant molecules perception pathway, we modified HPMA with thiol groups that bind tightly to ORs and further constructed thiol‐presenting nanoscale structures for drug delivery to the CNS, named nanoodors. Nanoodors exhibit a strong affinity for ORs, enhancing the brain accumulation of hydrophobic drugs in direct proportion to the thiol density on their surface. The nanoodors had a particle size of 140–170 nm and a hydrophobic core that encapsulated AGO, featuring a uniform dispersion and demonstrating a high encapsulation efficiency and slow drug release. In vivo studies indicated that the nanoodors rapidly accumulate in the brain, showing accumulation and antidepressant effects exceeding those of commercially available oral formulations.

Additionally, we elucidated the brain‐targeting delivery pathways and transport mechanisms of the nanoodors using various technical methods and identified the olfactory pathway as the most critical route. The nanoodors can rapidly delivered hydrophobic drugs to the CNS via the olfactory pathway. This study represents notable progress in the creation of effective materials for drug delivery to the brain. These materials have potential for use in therapeutic settings and can significantly benefit human health. In the future, the design of nanoodors will hopefully realize the efficient delivery and targeting of various brain disease drugs.

## 4 Experimental Section

### Materials

Agomelatine (AGO, 98%) and Cyanine7 amine (Cy7) were obtained from Aladdin Chemical Inc. (Shanghai, China). Cyanine2 (Cy2) was purchased from Yuanye Co., Ltd. (Shanghai, China). Antifade mounting medium containing DAPI was obtained from Dalian Meilun Biotech Co., Ltd. (Liaoning, China). Tissue‐TekO.C.T. Compound was purchased from Sakura Global Holding Co., Ltd. (Hong Kong, China). Phosphate‐buffered saline (PBS) was obtained from Wuhan Servicebio Technology Co., Ltd. (Wuhan, China). Ethanol was purchased from Sigma‐Aldrich (St. Louis, MO, UK). Amicon Ultra 4 mL centrifugal filter units (molecular weight cutoff (MWCO) = 3 kDa) were purchased from Merck Millipore (Massachusetts, USA). The olfactory neuronal marker OMP antibody (ab183947) was purchased from Abcam Co., Ltd. (Berlin, Germany). The Polyclonal MOR256‐17 Antibody was purchased from Novus Co., Ltd. (Missouri, USA). All other reagents were purchased from Shan Dong Yu Wang Reagent Co. (Shandong, China)

### Animals

Healthy Sprague–Dawley rats (6–8 weeks old) were purchased from Liaoning Changsheng Biotechnology Co., Ltd. (Liaoning, China) and housed in a pathogen‐free, light‐cycled, and temperature‐controlled facility. Sheep were sourced from local abattoirs. All animal experiments were performed in accordance with the guidelines approved by the Institutional Animal Use Committee of Shenyang Pharmaceutical University and the National Research Council's Guide for the Care and Use of Laboratory Animals (SYPU‐IACUC‐S2024‐0110‐201).

### Synthesis of pHPMA and pHPMASH

pHPMA is a copolymer composed of two monomers, namely, HPMA and *N*‐methacryloyl glycylglycine (MA‐GG‐OH).^[^ ^39^ ^]^ These monomers were synthesized using procedures described in previous papers.^[^ ^40^ ^]^ During the synthesis of pHPMASH, MA‐GG‐OH was replaced with thiolated‐*N*‐methacryloyl‐glycylglycine (MA‐GG‐SH), which was a newly derived compound achieved by attaching thiol groups to the MA‐GG‐OH segment through grafting. copolymer derivatives of pHPMASH were produced using radical solution polymerization in absolute methanol, at HPMA monomers at a ratio of 85:90:95 (Figure 2a). The resulting polymers were termed pHPMASH15, pHPMASH10, and pHPMASH5, respectively.^[^ ^41^ ^]^ Copolymerization was performed within sealed ampules under a nitrogen at a 50 °C for 24 h. pHPMASH was isolated through precipitation in diethyl ether, purified by dialysis, and lyophilized.

### Synthesis of AGO‐loaded nanoparticles

AGO‐loaded nanoparticles were prepared by rotary evaporation. AGO and pHPMA/pHPMASH were dissolved in ethanol, and the solution was agitated at room temperature for 10 min. This film was continuously hydrated with PBS (2 mL) at room temperature until full hydration was achieved (Figure 2b). Unentrapped AGO was removed using ultracentrifuge tubes. The liquid was loaded to Amicon ultra‐4 mL centrifugal filters (3 kDa MWCO) and spun at 5000 g for 25 min (HSC‐3020L, SCIENTZ, Zhejiang). The AGO‐loaded nanoparticles were collected from the sample reservoir of the filtration unit using a pipette tip. The purified dispersion was passed through a 0.22 µm microporous filter to remove microparticulate impurities and obtain a filtrate containing pure AGO‐loaded nanoparticles. The four types of nanoparticles were named pHPMA‐AGO, pHPMASH5‐AGO, pHPMASH10‐AGO, and pHPMASH15‐AGO.

### Preparation of Cy7‐ and Cy2‐loaded nanoparticles

The Cy7‐ and Cy2‐loaded nanoparticles were prepared using the rotary evaporation method. The Cy7/Cy2 and pHPMASH/pHPMA were dissolved in ethanol. The mixture was agitated at ambient temperature for 10 min, and ethanol was evaporated at 35 °C under vacuum using a rotary evaporator. The thin film formed was hydrated with PBS and the bottle was shaken continuously at room temperature until the film formed was fully hydrated. Free Cy7/Cy2 was removed using ultracentrifuge tubes. The liquid was loaded to Amicon ultra‐4 ml centrifugal filters (3 kDa MWCO) and spun at 5000 g for 25 min (HSC‐3020L, SCIENTZ, Zhejiang). The concentrate was collected from the sample reservoir of the filtration unit using a pipette and washed with PBS until the filtrate became colorless. The purified dispersion from the sample reservoir of the filtration unit was passed through a 0.22 µm microporous filter to remove microparticulate impurities and obtain a filtrate containing Cy7‐/Cy2‐loaded nanoparticles.

### Characterization

The particle size distributions, polydispersity (PDI), and ζ‐potentials of AGO‐loaded nanoparticles were measured using dynamic light scattering (DLS) (Nano‐ZS90 nanosizer, Malvern Instruments, UK). The morphology and mesoporous nanostructure of AGO nanoparticles were observed using transmission electron microscopy (TEM) (Tecnai G2 F30, FEI, Netherlands).

### Determination of AGO encapsulation efficiency

The AGO‐loaded nanoparticles were fixed in ethanol (2 mL) and ultrasonicated at 400 W for 15 min to extract AGO from the core of the nanoparticles. The resulting solutions were filtered through a 0.45 µm membrane, and the absorbance of the filtrate at 303 nm was measured using an UV–visible spectrophotometer (UV‐2150, UNICO, US). The drug loading (DL%) and drug encapsulation efficiency (EE%) were calculated with Equations (1) and (2), respectively.

(1) $$\mathrm{DL}\;\left(\%\right)=\frac{W_{\mathrm{encapsulated}}}{W_{\mathrm{nanoparticles}}}\times 100$$

(2) $$\mathrm{EE}\;\left(\%\right)=\frac{W_{\mathrm{encapsulated}}}{W_{\mathrm{total}}}\times 100$$

where *W*_encapsulated_, *W*_total_, and *W*_nanoparticles_ represented the weight of AGO encapsulated in the nanoparticles, the AGO total weight, and the weight of AGO‐loaded nanoparticles, respectively.

### In vitro drug release behaviors

In vitro drug release behavior was studied using the dialysis method.^[^ ^42^, ^43^ ^]^ A sample containing 4 mg of the drug was loaded into a dialysis bag (3.5 kDa MWCO) and dialyzed against artificial CSF (10 mL) in a thermostatic oscillator (37 °C, 200 rpm). After 0.5, 1, 2, 4, 6, and 8 h, 2 mL of the dialysis medium was withdrawn and replaced with fresh medium to maintain sink conditions. The concentration of the drug in each sample was determined based on absorbance at 303 nm.

### Stability of AGO‐loaded nanoparticles

Formulation stability was assessed by quantifying the evolution of particle size over three days. The effect of the delivery technique on particle size was evaluated by introducing the AGO‐loaded nanoparticles into a nasal spray device and comparing particle dimensions before and after spraying. An RST‐CPS‐FH viscometer (Brookfield, USA) was used to examine the effect of shear stress on viscosity in the four groups of the AGO‐loaded nanoparticles.

### Transmembrane experiments

Olfactory mucosa was harvested from fresh sheep heads. A Franz diffusion cell was used. Physiological saline (14 mL) was added to the receiving chamber, and air bubbles were removed in a timely manner. The nasal mucosa layer was immobilized between the receiving and supplying chambers, positioned on a water bath held at 37 °C, and allowed to stabilize for 30 min. Subsequently, Cy7‐loaded nanoparticles (200 µL) were introduced into the supplying chamber. The nasal mucosa was removed after 4 h, washed, and incubated in a 4% paraformaldehyde solution for 24 h. The segments were preserved by freezing in a cryoembedding medium, cut into 10 µm thick sections, and mounted onto glass slides. Confocal microscopy observations of nasal mucosa tissues were conducted to investigate the transmembrane characteristics of Cy7‐loaded nanoparticles. Simultaneously, physiological saline was withdrawn from the receptor pools of each group, and 200 µL aliquots were transferred to a 96‐well plate. The fluorescence intensity of each group was measured using a multifunction microplate reader (Varioskan Flash; Thermo Scientific) as a measure of the ability of each nanoparticle type to permeate the olfactory mucosa.

### Optical imaging of rats

Cy7‐loaded nanoparticles solution and Cy7 solution were administered intranasally to SD rats at a dose of 100 µL. Scans were conducted at 1, 2, and 4 h postadministration. At 4 h postadministration, major organs, including the brain, heart, liver, lungs, spleen, and kidneys, were rapidly excised and immediately subjected to ex vivo imaging (*n* = 3). All images were analyzed using the Living Image 4.3.1 software (Caliper Life Sciences). During imaging, the animals were sedated with a mixture of 1.5% isoflurane and 98.5% oxygen.

### Pharmacokinetic analysis

Seventy‐five rats were randomly divided into five groups. Four groups were intranasally treated with a solution of AGO‐loaded nanoparticles (100 µL), and the remaining group was orally treated with the AGO suspension. CSF, plasma, and brain tissues were collected from each group at 0.25, 0.5, 1, 3, and 6 h postadministration. Blood samples (0.25 mL) were drawn from the orbit, transferred into 1.5 mL centrifuge tubes prerinsed with heparin sodium solution, and centrifuged at 1500 g for 15 min at 4 °C to collect the supernatant. CSF samples were extracted from the cisterna magna and centrifuged at 3000 rpm for 15 min at 4 °C to collect the supernatant. Whole brain samples were harvested, and saline was added to the tissues in a ratio of 1:2 (v/v, tissue/saline). After processing, the concentration of AGO in the samples was quantified using high‐performance liquid chromatography‐tandem mass spectrometry. The mobile phase (methanol‐0.3% formic acid‐10 mM ammonium acetate–water) was supplied at a flow rate of 0.30 mL min^−1^. Pharmacokinetic data were derived from the area under the curve (AUC). Pharmacokinetic parameters were derived from drug concentration–time data with DAS 2.0 software. The ratio AUC_in_/AUC_pHPMA_ is represented using Equation (3).

(3) $$\mathrm{AUCin}/\mathrm{AUCpHPMA}=\frac{\mathrm{AUCpHPMASH}\;\mathrm{nanoparticles}}{\mathrm{AUCpHPMA}\;\mathrm{nanoparticles}}$$

### Histopathology studies

The rat nasal mucosa was isolated after drug administration. The nasal mucosa was subjected to isopropanol treatment as a positive control,^[^ ^44^, ^45^ ^]^ whereas saline solution treatment was used as a negative control. The nasal mucosa was embedded in paraffin, sectioned, and stained with hematoxylin and eosin. After dehydration and sealing, the sections were observed under a microscope and photographed for analysis.

### Pharmacodynamic analysis

Antidepressant effects were assessed using the modified forced swim test (MFST).^[^ ^46^, ^47^ ^]^ Among the developed carriers, pHPMASH15‐AGO nanoparticles targeted the brain most effectively and were therefore selected for further pharmacological testing. The complete 5‐min swim test was evaluated in rats.^[^ ^48^ ^]^ The despair degree was evaluated by determining the percentage of immobile time in water during the test period.^[^ ^49^ ^]^ The procedure began with a 15 min swimming session (preswim), which was conducted 24 h before the main 5 min test. This preswim session helped stabilize immobility levels, facilitating the detection of antidepressant activity. Subsequently, the rats were removed from water, dried with towels, and returned to their cages. The rats received three administrations of the drugs or the appropriate vehicle control 1, 5, and 23.5 h before the swim tests. Scoring was based on two types of active behaviors, swimming and climbing, and the use of a time sampling technique that measured the predominant behavior is measured in 5 s intervals. Swimming was defined as vigorous motions going beyond merely keeping the head above the water, whereas climbing was defined as activities involving energetic forepaw movements typically directed against the tank walls. Thirty rats were randomly divided into five groups (*n* = 6), and the extreme value of the result was removed:

Group I: control group received PBS by intranasal administration.
Group II: standard group received the AGO suspension orally (p.o.) (2.5 mg kg^−1^).
Group III: medium dose group received the formulation by intranasal administration (i.n.) (2.5 mg kg^−1^).
Group IV: high dose group received the formulation by intranasal administration (i.n.) (3.75mg kg^−1^).
Group V: low dose group received the formulation by intranasal administration (i.n.) (0.75mg kg^−1^)

### Evaluation of olfactory mucosal epithelial route

The Franz diffusion cell was used. Physiological saline (14 mL) was added to the receiving chamber of the Franz diffusion cell, and air bubbles were discharged in a timely manner. The sheep nasal mucosa was divided into nine sections. The mucosa layer was secured between the receiving and supplying chambers and incubated in a water bath held at 37 °C for 30 min. Subsequently, 200 µL of Cy7 nanoparticles were added into the supplying chambers. Sections were removed at 0.5, 1, and 60 min and immersed in 4% paraformaldehyde for 24 h. The segments were preserved by freezing in a cryoembedding medium, cut into 10 µm thick sections, and mounted onto glass slides. The transmembrane transport of Cy7‐loaded nanoparticles across the olfactory epithelia was observed by confocal microscopy.

### Nanoodors bind to olfactory receptors

Nasal mucosa from rats collected at 1 h postadministration were processed into paraffin sections for immunofluorescence staining. The OR gene MOR256‐17 (also known as Olfr15, OR2C1 and OR3) receptor exhibits the broadest odorant response profile and can react with various compounds, with a particularly strong interaction with thiol compounds.^[^ ^50^ ^]^ We used a MOR256‐17 antibody to label MOR256‐17, with Cy5 as the secondary antibody (yellow), whereas pHPMASH15 loaded with Cy2 was used to present green fluorescence.

### Drug distribution in olfactory receptor neurons and olfactory nerve

Nasal tissues from rats collected at 1 h postadministration were processed into paraffin sections for immunofluorescence staining. OMP antibodies conjugated with Cy3, which emits red fluorescence, were used to target the ORNs and olfactory nerves. pHPMASH15 nanoparticles loaded with Cy2, which emits green fluorescence, were used for imaging. Using confocal microscopy, we observed the colocalization of the pHPMASH15‐Cy2 nanoparticles with OMP antibody‐labeled olfactory neurons in the olfactory region of the nasal mucosa.

### 3D structures of nose and brain tissues in rats

Brain and nasal tissues from rats were collected and subjected to immunofluorescence staining using Cy5 as a secondary antibody. The 3D structure observed using Cy2 revealed the presence of the poorly soluble drug (green) and olfactory marker protein (OMP) marking ORNs and olfactory nerves (yellow). This approach was used to verify the presence of the olfactory nerve route. After the immunofluorescence staining of all 80 consecutive sections, they were scanned consecutively, and the scanned images were stacked to form a 3D stereogram using the 3DView software. The colocalization of Cy2, which was substituted for the drug, with the ORNs, as well as its distribution in the brain tissues, were observed.

### Statistical analysis

Results are presented as mean ± standard deviation (SD). Groups were analyzed using one‐way analysis of variance (ANOVA) with an appropriate post‐hoc test (Tukey's). Statistical significance was set as *P < 0.05, **P < 0.01, ***P < 0.001, ****P < 0.0001 (ns: no significance), with a 95% confidence interval. The GraphPad Prism v.10.0 and Origin programs were used for analysis and figure generation.

## Conflict of Interest

The authors declare no conflict of interest.

## Supporting information



Supporting Information

## Data Availability

The data that support the findings of this study are available from the corresponding author upon reasonable request
