# Supplementary material for: Nanoodor Particles Deliver Drugs to Central Nervous System via Olfactory Pathway
Source: Adv Sci (Weinh). 2025 Feb 25;12(15):2408908. doi: 10.1002/advs.202408908 (PMC12005826; doi:10.1002/advs.202408908)
Supplement: Supplementary file 1 — Supporting Information [file ADVS-12-2408908-s001.docx]

**Supporting Information**

**NanoOdor Particles Deliver Drugs to the Central Nervous System via Olfactory Pathway**

Wei Zhang^a^, Xingwang Ji^a^, Qianyanqiu Zhao^a^, Jinyao Qi^a^, Wen Guo^a^, Gaoshuo Zhang^a^, Yujing Guan^d^, Shenglong Li^b,c,d*^, Yuling Mao^a*^

^a^ Department of Pharmaceutics, School of Pharmacy, Shenyang Pharmaceutical University, 103 Wenhua Road, Shenyang, Liaoning Province 110016, P.R. China

^b^ Second Ward of Bone and Soft Tissue Tumor Surgery, Cancer Hospital of Dalian University of

Technology, Cancer Hospital of China Medical University, Liaoning Cancer Hospital & Institute,

Shenyang, Liaoning Province, 110042, China

^c^ The Liaoning Provincial Key Laboratory of Interdisciplinary Research on Gastrointestinal Tumor Combining Medicine with Engineering, Shenyang, Liaoning Province, 110042 China

^d^ Institute of Cancer Medicine, Faculty of Medicine,Dalian University of Technology, No.2 Linggong Road, Ganjingzi District, Dalian, 116024, Liaoning Province, China

**The corresponding author***

Yuling Mao

1. mail address: [maoyuling@syphu.edu.cn; maoyuling@hotmail.com](mailto:maoyuling@syphu.edu.cn，maoyuling@hotmail.com)

Shenglong Li

E-mail address：slli@cmu.edu.cn; [lishenglong@cancerhosp-ln-cmu.com;](mailto:lishenglong@cancerhosp-ln-cmu.com;) lishenglong@dlut.edu.cn

**Table S1** Shorthand for HPMA modified with different proportions of thiol groups.

| Sample Name | Free molar ratio |
| --- | --- |
| pHPMASH5 | HPMA/MA-GG-SH=95:5 |
| pHPMASH10 | HPMA/MA-GG-SH=90:10 |
| pHPMASH15 | HPMA/MA-GG-SH=85:15 |

**Table S2** The encapsulation efficiency (EE), drug loading (DL) of self-assembled nanoparticles respectively.

|  | Entrapment efficiency (EE) (%) | | | Drug loading (DL) (%) | | |
| --- | --- | --- | --- | --- | --- | --- |
| pHPMA-AGO | 81.6 | 84.1 | 82.8 | 7.41 | 7.65 | 7.53 |
| pHPMASH5-AGO | 76.8 | 81.8 | 78.0 | 6.97 | 7.44 | 7.09 |
| pHPMASH10-AGO | 83.7 | 84.5 | 82.4 | 7.61 | 7.68 | 7.49 |
| pHPMASH15-AGO | 79.9 | 83.4 | 82.2 | 7.26 | 7.58 | 7.47 |


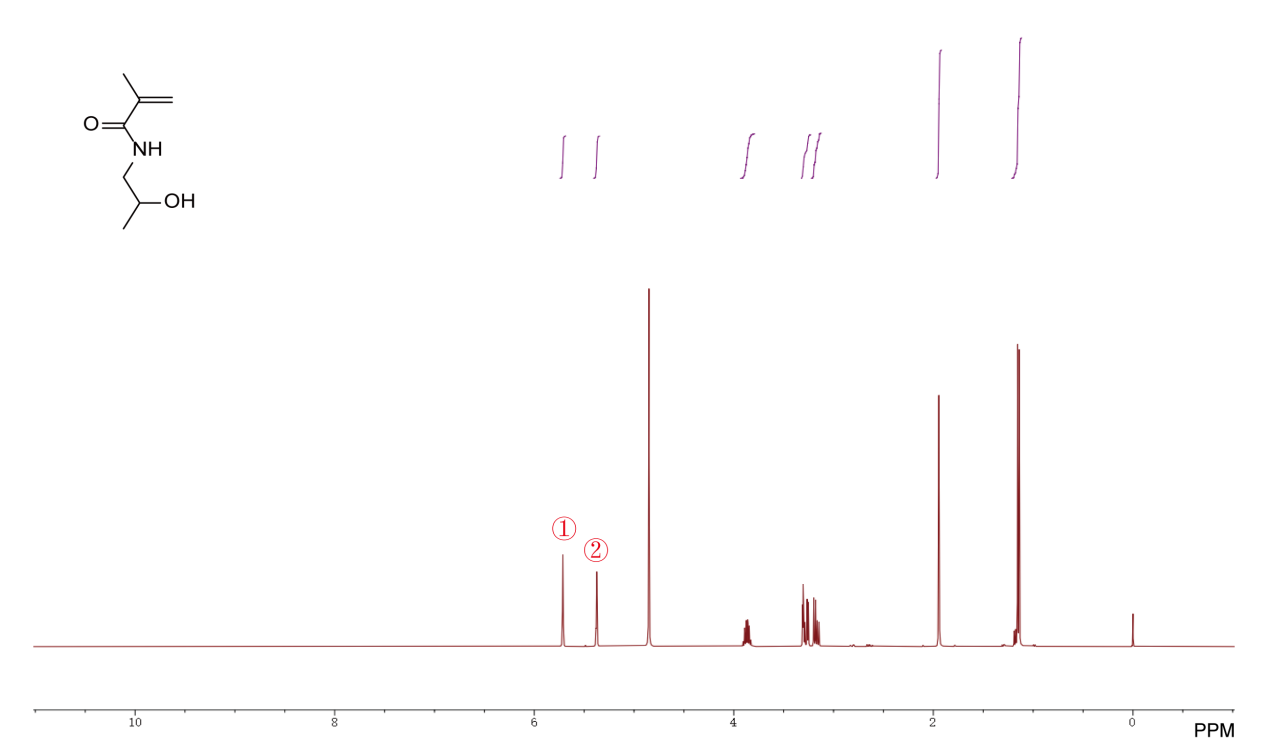


**Figure S1** The ^1^H-NMR (MeOD) spectrum of HPMA monomer.

**
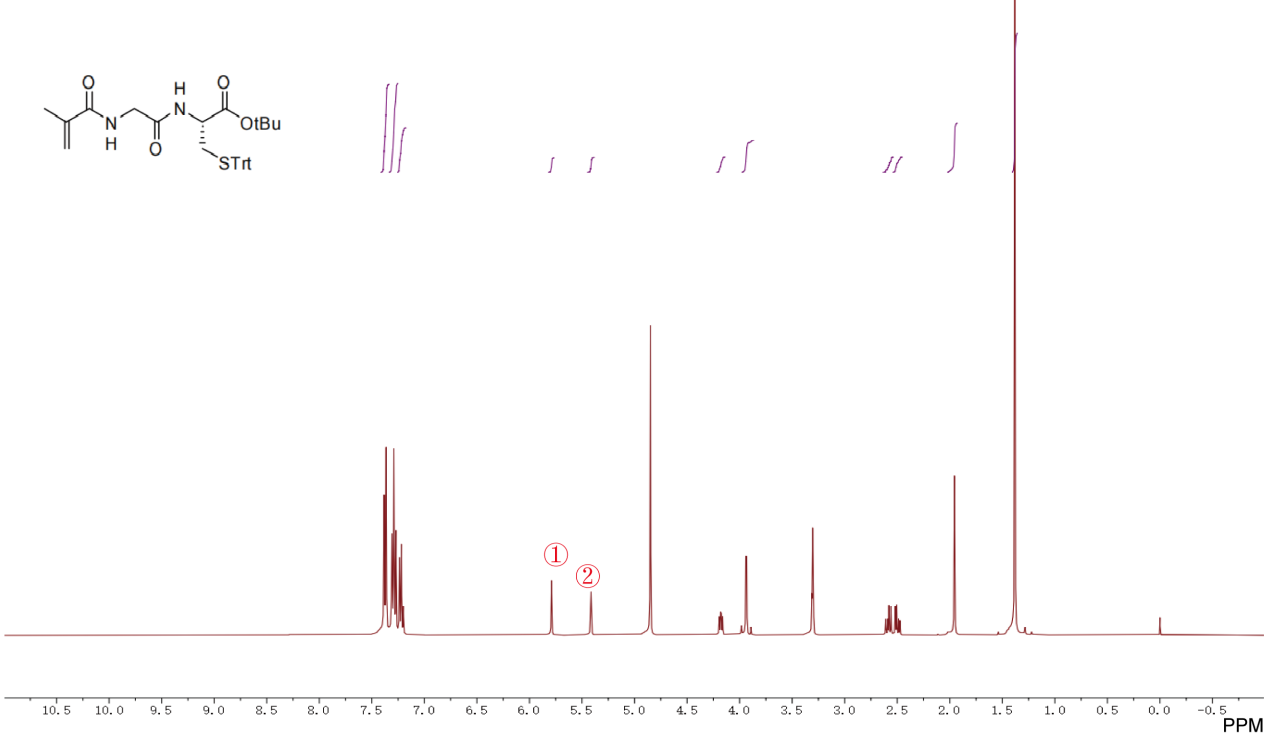
**

**Figure S2** The ^1^H-NMR (MeOD) spectrum of MA-GG-SH monomer.


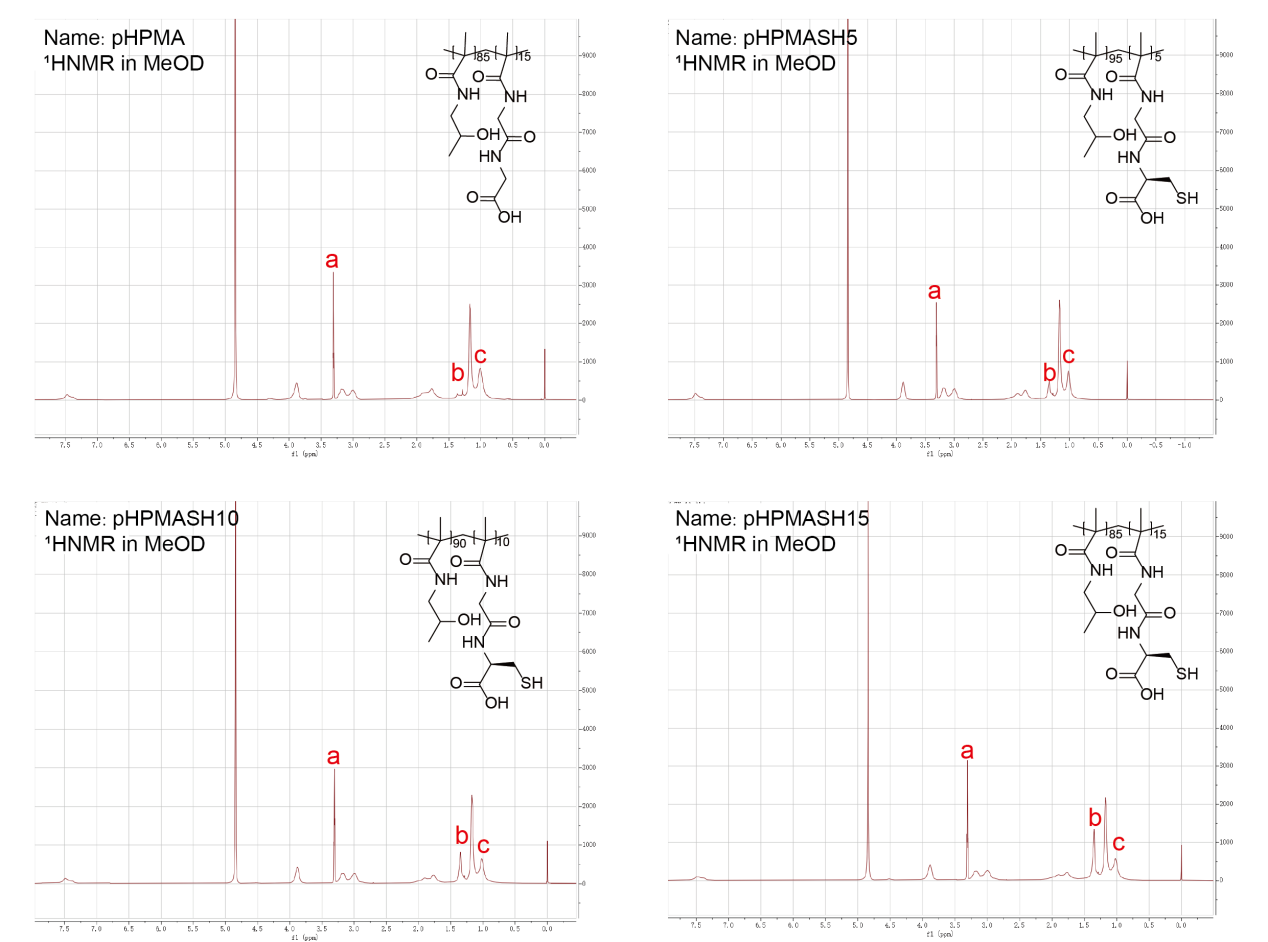


**Figure S3** The ^1^H-NMR (MeOD) spectrum of pHPMA, pHPMASH5, pHPMASH10, pHPMASH15 derivative. Compared with the ^1^H-NMR spectra of HPMA and MA-GG-SH monomers, it is indicated that all of the resonances from the alkenyl groups disappeared after polymerization. From the ^1^H-NMR (MeOD) spectrum of pHPMA, pHPMASH5, pHPMASH10, and pHPMASH15 derivatives, it is evident that thiol groups were successfully grafted onto pHPMA, resulting in polymers with varying proportions of thiol modifications.


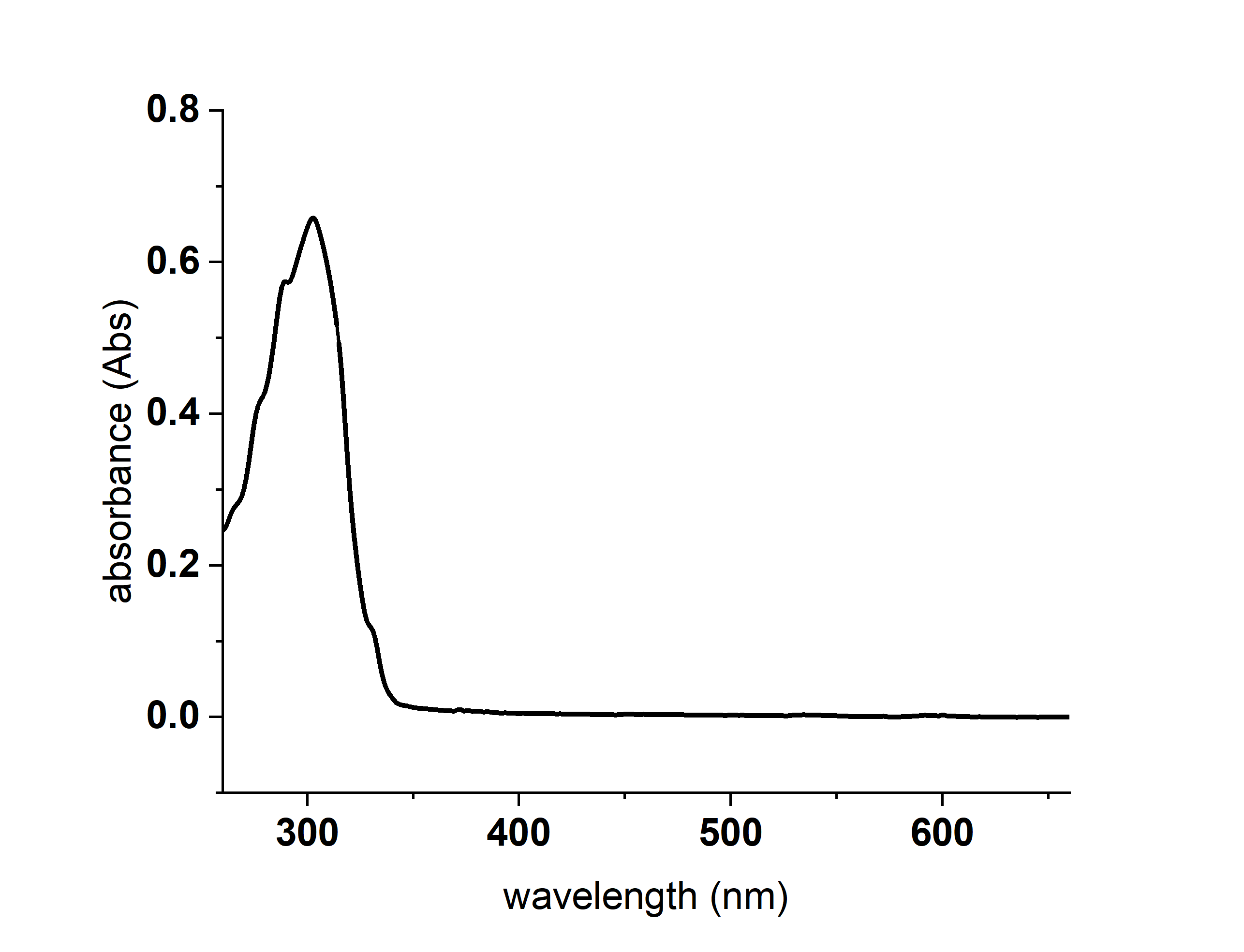


**Figure S4** The relationship between agomelatine absorbance and wavelength.

**
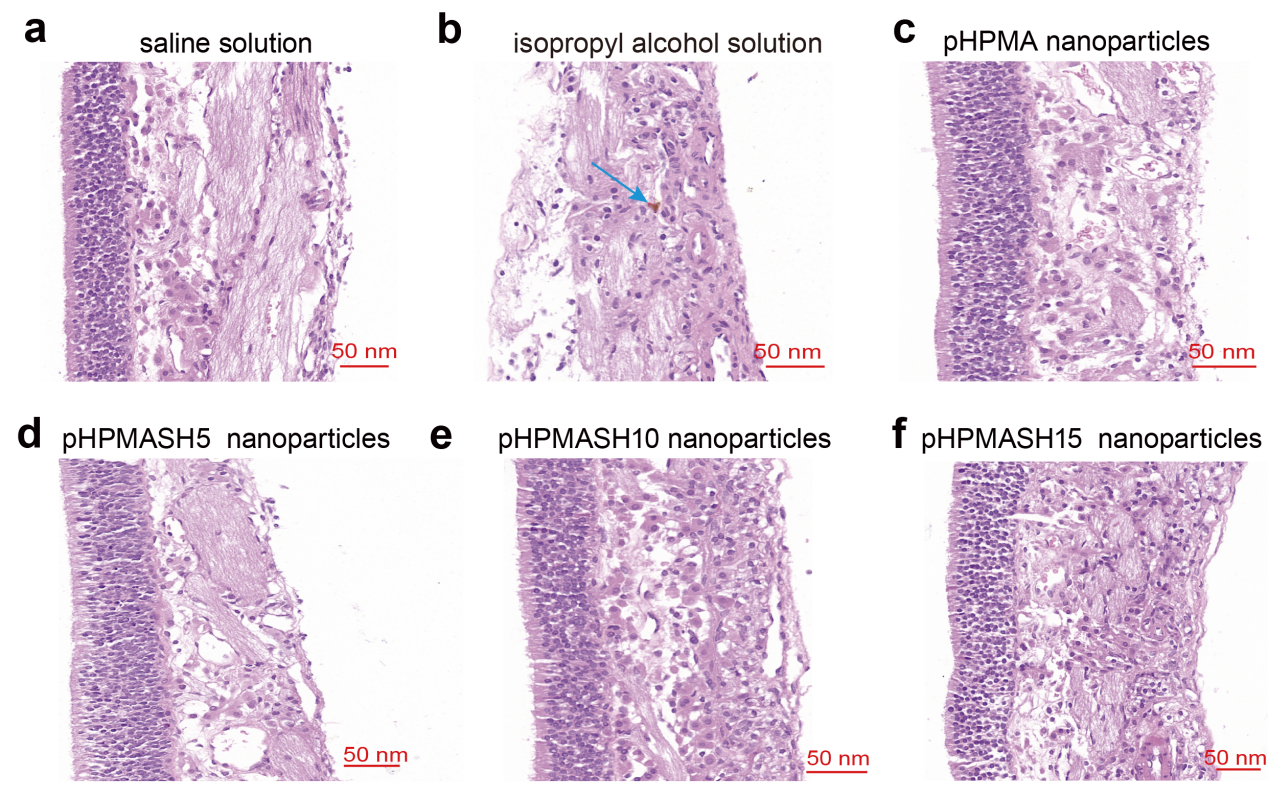
**

**Figure S5** H&E stains to show the irritant of different nanoparticles through the tissue morphology. a) nasal mucosa treated with saline solution was used as a negative control and b) Nasal mucosa treated with isopropanol was used as a positive control. c) H&E stains results of pHPMASH nanoparticles, d) pHPMASH5 nanoparticles, e) pHPMASH10 nanoparticles, and f) pHPMASH15 nanoparticles.

**
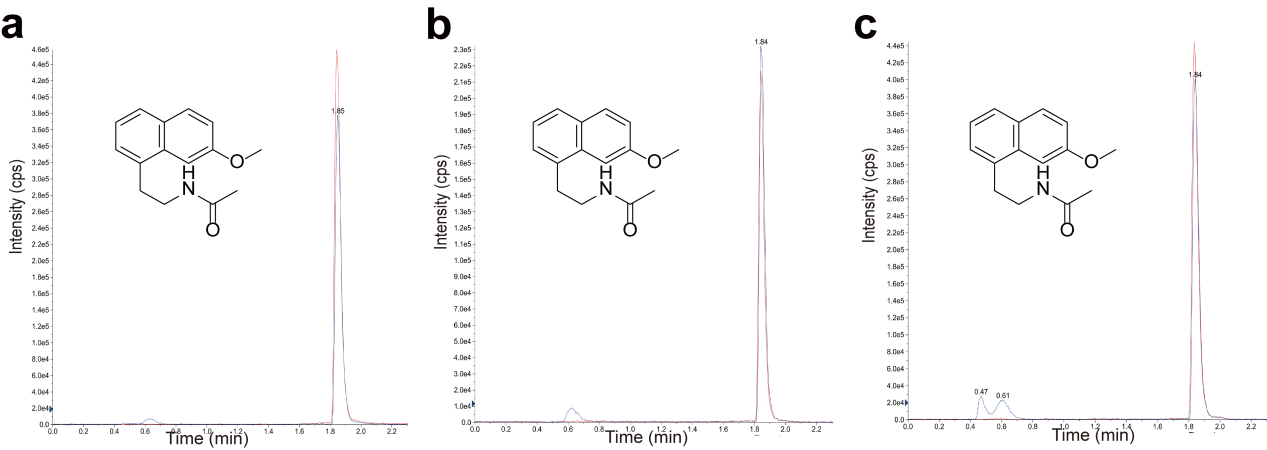
**

**Figure S6** a) In cerebrospinal fluid, b) blood and brain tissue, c) representative maps of Agomelatine.

**
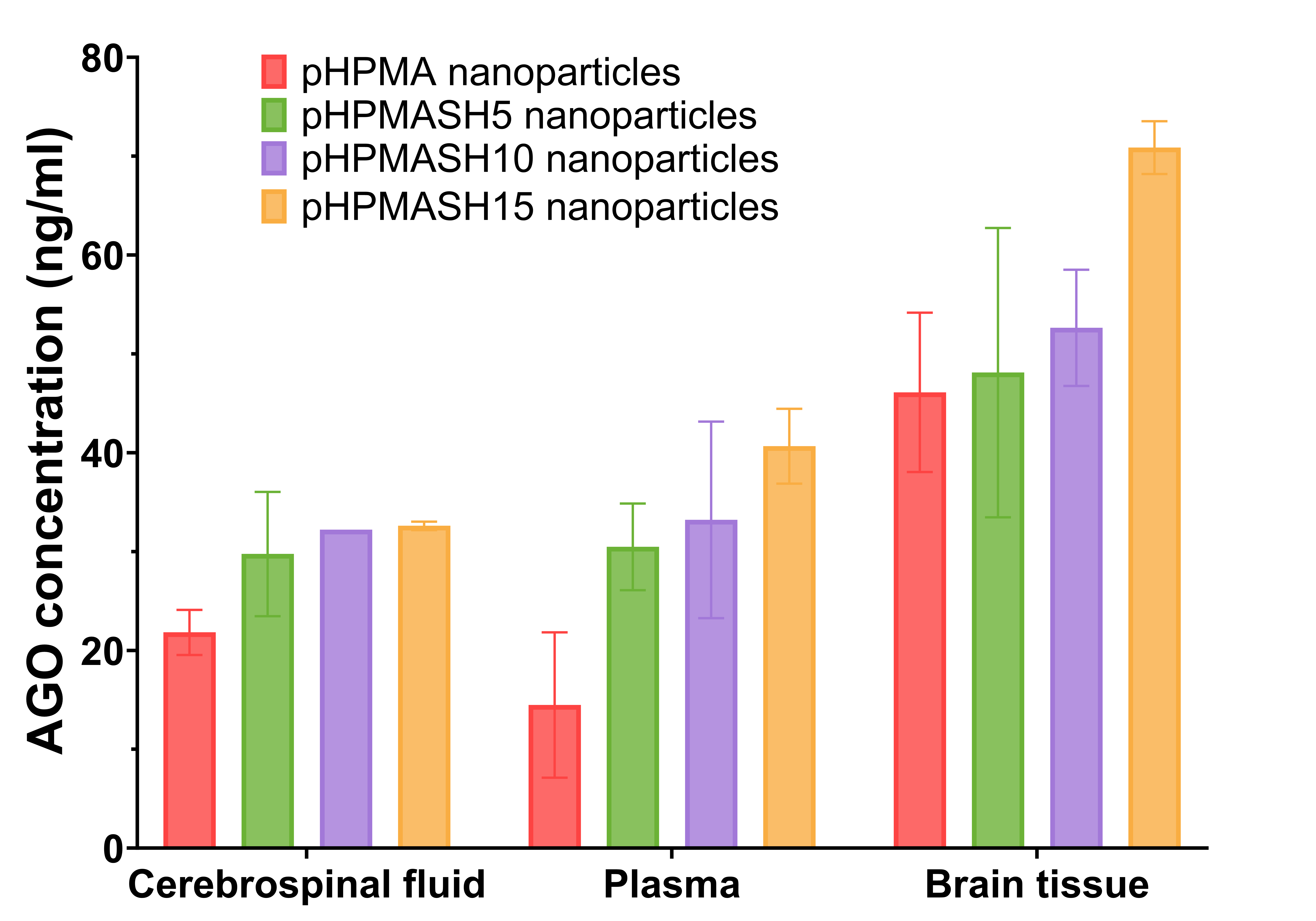
**

**Figure S7** Maximum cerebrospinal fluid, plasma, and brain tissues concentrations of AGO in rats after intranasal administering.


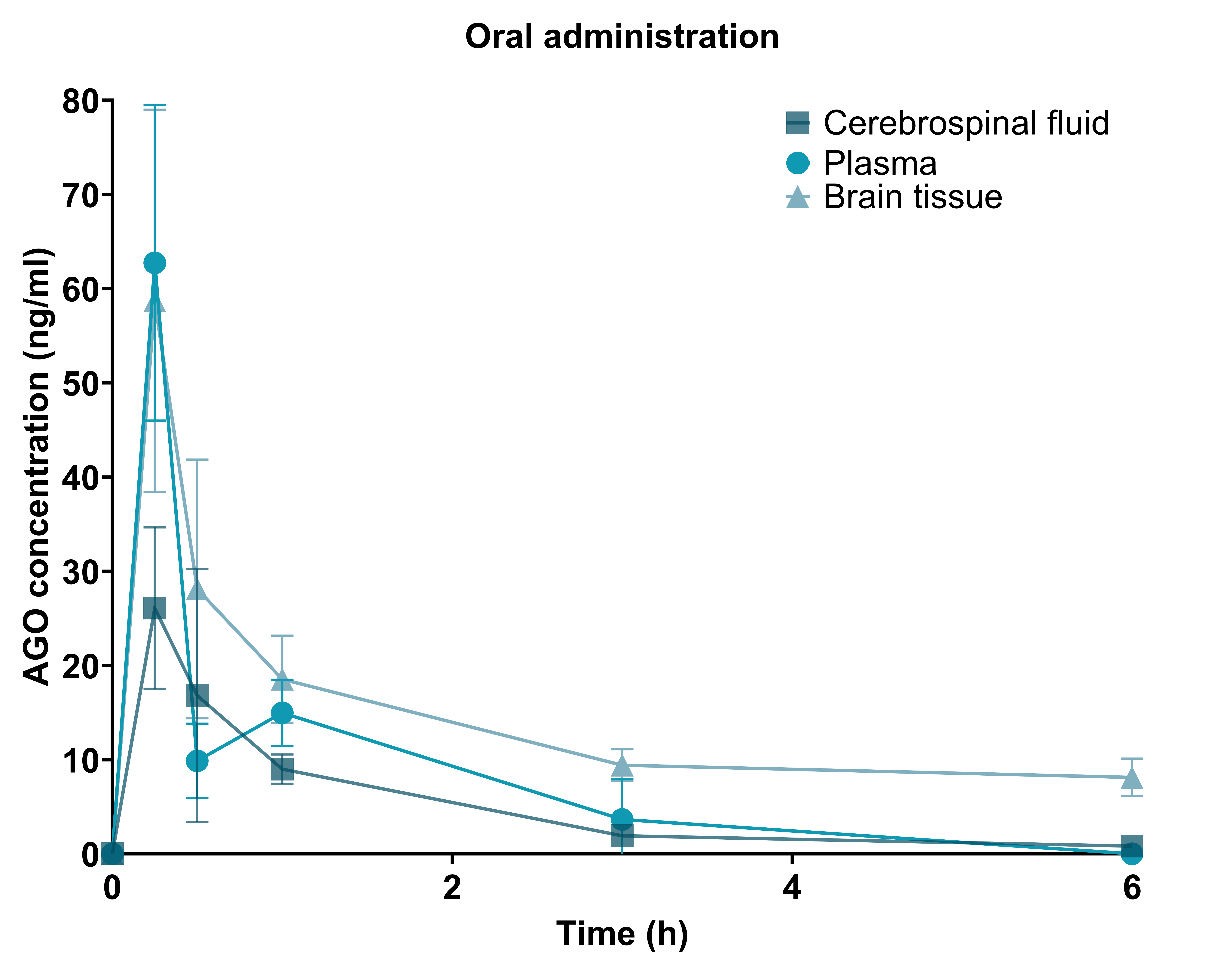


**Figure S8** Cerebrospinal fluid, plasma and brain tissues concentrations of AGO in rats after orally administering formulations at dose of equal to AGO at 0.25h, 0.5h, 1h, 3h and 6h.

**Table S3** The AUC_Brain_, AUC_CSF_, C_max_-Brain, C_max_-CSF of pHPMA-AGO, pHPMASH5-AGO, pHPMASH10-AGO and pHPMASH15-AGO groups by intranasal administration and AGO solution by oral administration.

|  | Average value | | | |
| --- | --- | --- | --- | --- |
|  | AUC_Brain_  /（ng·h/ml） | AUC_CSF_  /（ng·h/ml） | C_max_-Brain  /(ng/ml) | C_max_-CSF  /(ng/ml) |
| AGO (p.o.) | 77.32 | 32.26 | 22.10 | 47.00 |
| pHPMASH-AGO (i.n.) | 107.83 | 38.82 | 46.10 | 21.83 |
| pHPMASH5-AGO (i.n.) | 122.76 | 45.29 | 48.10 | 29.75 |
| pHPMASH10-AGO (i.n.) | 125.59 | 45.45 | 52.63 | 32.20 |
| pHPMASH15-AGO (i.n.) | 132.86 | 48.14 | 70.87 | 32.60 |

**
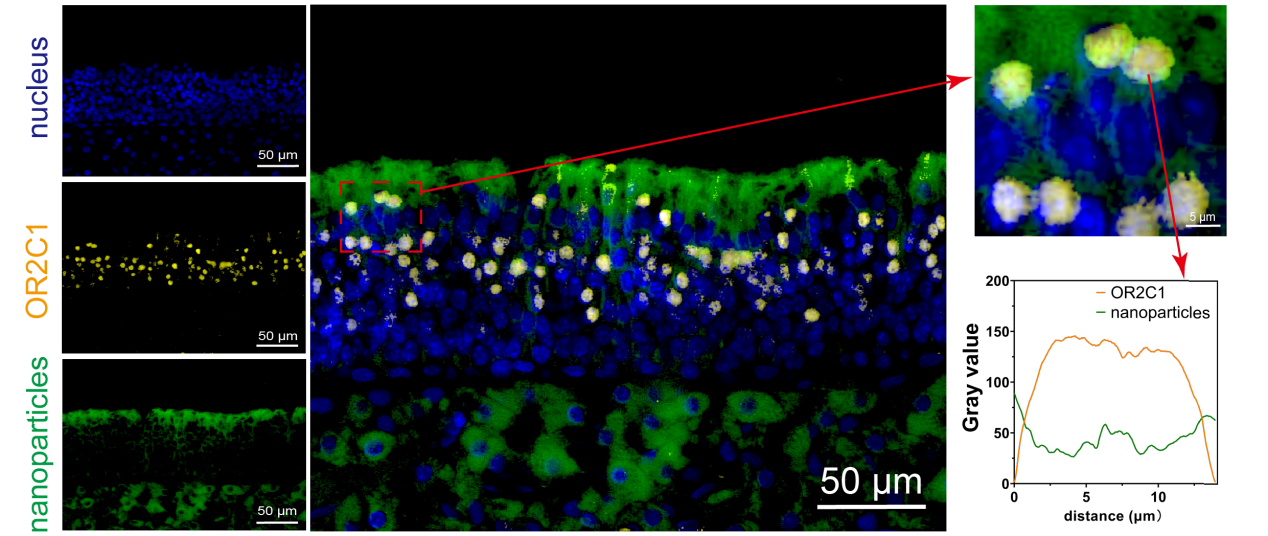
**

**Figure S9** Nanoodors interact with olfactory receptor. An immunofluorescence dual staining image showing nanoodors (green) and the olfactory receptor (yellow).

**
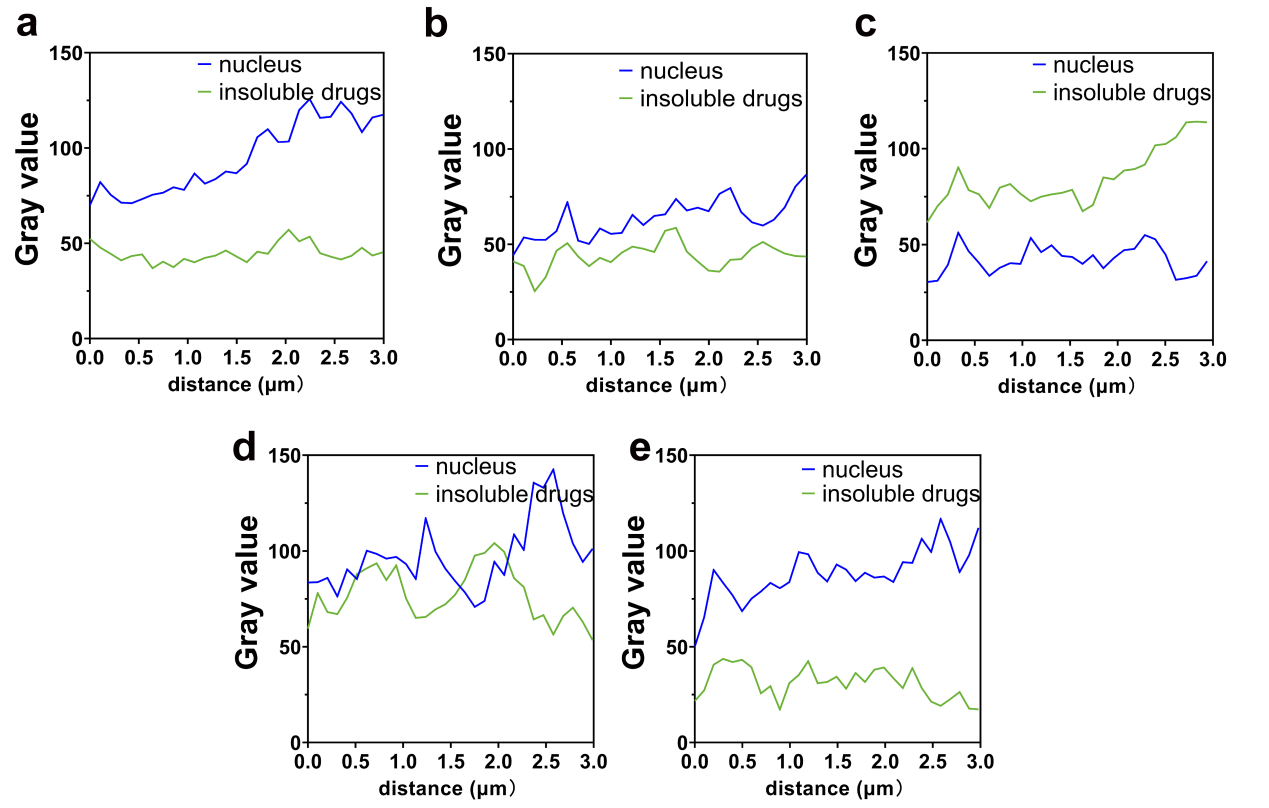
**

**Figure S10** Distribution of insoluble drugs in the brain. Segmentation at five sites: a) olfactory bulb, b) frontal cortex, c) ventral tegmental area, d) dorsal raphe nucleus, and e) locus coeruleus.
